# Predicting 30-day survival after in-hospital cardiac arrest: a nationwide cohort study using machine learning and SHAP analysis

**DOI:** 10.1136/bmjopen-2024-090493

**Published:** 2025-04-27

**Authors:** Vibha Gupta, Aidin Rawshani, Peter Lundgren, Mats Börjesson, Truls Råmunddal, Per Nordberg, Therese Djärv, Johan Herlitz, Joakim Sundström, Carl Magnusson, Antros Louca, Erik Andersson, Kristofer Skoglund, Araz Rawshani

**Affiliations:** 1 Department of Molecular and Clinical Medicine, Institute of Medicine, University of Gothenburg, Gothenburg, Sweden; 2 Wallenberg Center for Molecular and Translational Medicine, Wallenberg Laboratory, University of Gothenburg, Goteborg, Sweden; 3 Centre for Digital Health, Område Digitalisering, Sahlgrenska University Hospital, Goteborg, Sweden; 4 Department of Cardiology, Sahlgrenska University Hospital, Goteborg, Sweden; 5 Department of Clinical Science and Education, Karolinska Institutet, Stockholm, Sweden; 6 Molecular Medicine and Surgery, Karolinska Institutet, Stockholm, Sweden; 7 University of Borås, Faculty of Caring Science, Work Life and Social Welfare, Borås, Sweden

**Keywords:** Cardiac Epidemiology, Death, Sudden, Cardiac, Defibrillators

## Abstract

**Abstract:**

**Objective:**

In-hospital cardiac arrest (IHCA) presents a critical challenge with low survival rates and limited prediction tools. Despite advances in resuscitation, predicting 30-day survival remains difficult, and current methods lack interpretability for timely decision-making. This study developed a machine learning (ML) model to predict 30-day survival after IHCA, using peri-arrest variables available on the rescue team’s arrival, while ensuring a balance between predictive accuracy and clinical interpretability through Shapley Additive Explanations (SHAP).

**Design:**

A nationwide, registry-based observational study.

**Setting:**

Data were sourced from the Swedish Cardiopulmonary Resuscitation Registry (2010–2020), merged with the Patient Registry.

**Participants:**

We analysed 25 905 IHCA cases with attempted resuscitation, of which 8166 patients survived for 30 days.

**Outcome measure and analysis:**

30-day survival after IHCA was the outcome measure. An ML model was developed using fivefold cross-validation. Key predictors were identified through in-built variable importance and validated using SHAP. Model performance was evaluated with metrics such as area under the receiver operating characteristics (AUROC), calibration, sensitivity, specificity, false negative rate (FNR) and F-score.

**Results:**

The CatBoost model achieved an AUROC of 0.9136 (95% CI 0.9075 to 0.9191) with all features, and 0.9034 (95% CI 0.8955 to 0.9037) with the top 15 features, along with Brier scores of 0.1028 and 0.1103, respectively. Performance plateaued after including the top 15 predictors, with few key variables, such as epinephrine administration, age, initial rhythm, ROSC within 15 min, breathing on rescue team arrival and witnessed cardiac arrest, being most influential. The model showed strong calibration for patients with low predicted survival probabilities and demonstrated high sensitivity with a low FNR across relevant survival thresholds.

**Conclusion:**

The CatBoost model provides an effective and interpretable tool for predicting 30-day survival after IHCA. Key predictors such as epinephrine administration, age and initial rhythm inform clinical decision-making. This model has strong clinical utility and can be externally validated via the open-access Application Programming Interface (API) at www.gocares.se

**Strengths and limitations of this study**

- In this study, we leveraged the in-built feature importance evaluation of CatBoost, offering key advantages over traditional methods.
- Use of peri-arrest variables enables timely predictions to guide clinical interventions.
- External validation through independent datasets is needed to determine generalisability, which can be done via the open-access API (www.gocares.se).
- Data imbalance was handled internally, but further work is required to address its impact.

## Introduction

 In-hospital cardiac arrest (IHCA) is a substantial clinical challenge worldwide. Since it can affect any hospitalised patient, hospital personnel are trained in basic and advanced life support. Moreover, resourced hospitals have dedicated rescue teams that respond promptly to cardiac arrest. Despite IHCA occurring within hospital walls, often being witnessed, and cardiopulmonary resuscitation (CPR) being initiated immediately, the survival rate remains around 35%.^1^ Every year, about 2200 patients suffer IHCA,^2^ with nearly 40% of these incidents occurring in regular wards, where healthcare staff quickly notice and respond. Most IHCA cases have a do-not-attempt-resuscitation (DNAR) order and are therefore not included in the register.^3^ The marked difference in survival between IHCA and out-of-hospital cardiac arrest (OHCA) is explained by the difference in time from collapse to resuscitation, including defibrillation. This was demonstrated in a recent Danish registry study which reported that patient demographics, comorbidities, etc were similar in OHCA and IHCA, and adjusting for these did not affect differences in survival whereas adjusting for resuscitation differences abolished the differences.^4^

Estimating the probability of survival in IHCA, preferably during the initial phases of resuscitation, is important for prognostication and to assist the clinician’s judgement. A total of 16 prediction models have been developed for IHCA, but only 6 have been validated using external data.^5^ The Good Outcome Following Attempted Resuscitation (GO-FAR) score,^6^ developed to assist in DNAR decisions, has demonstrated the highest performance, with an area under the receiver operating characteristics (AUROC) of 0.78 (95% CI 0.59 to 0.85).

For several reasons, we believe that there is substantial room for improvement in this area. Most earlier models use traditional logistic regression, frequently with univariate variable screening, which has significant drawbacks.^7^ There have been variations with regard to how pre- and peri-arrest variables are included and at what time-point variable inclusion should end for a model to be clinically applicable during the initial phase of resuscitation. A clinically useful model that can assist decision-making early in the course must demonstrate excellent discrimination and calibration. While discrimination measures how well the model distinguishes between different outcomes, calibration assesses the accuracy of predicted probabilities compared with actual outcomes. Of utmost importance is the discrimination and calibration of the probability range where decisions are ambiguous, which, for IHCA, would be the lower half of the probability interval.

Our study addressed critical gaps in predicting 30-day survival following IHCA by developing an advanced machine learning (ML)-based prediction model. By focusing on the crucial time window when resuscitation decisions were made, our model integrated key peri-arrest variables available on the arrival of the rescue team. This approach ensured that predictions were based on clinically relevant, real-time data, delivering immediate and actionable insights to support clinical decision-making. We incorporated state-of-the-art ML algorithms such as Extreme Gradient Boosting (XGBoost),^8^ Categorical Boosting (CatBoost)^9^ and Light Gradient Boosting Machine (LightGBM),^10^ all known for their ability to handle complex interactions, missing data and categorical variables without extensive preprocessing. These algorithms also excelled in situations with imbalanced datasets, a common issue in clinical data.^11 12^ A key strength of these models was their ability to provide feature importance rankings, which could be leveraged to streamline the prediction process. This allowed for faster decision-making with a reduced number of highly relevant predictors, optimising both accuracy and efficiency.

To enhance the model’s interpretability, an often-challenging aspect of ML, we used Shapley Additive Explanations (SHAP). SHAP not only provided insights into individual feature contributions but also improved transparency, making the model more understandable and clinically applicable. By incorporating both advanced predictive power and clear interpretability, our model offered a balanced approach to decision-making in critical care settings. To ensure the model’s broad accessibility and facilitate independent validation, it was deployed with an open API (Swedish cardiac arrest risk score (SCARS)-2), enabling its use and evaluation by clinicians and researchers worldwide. With a focus on clinical utility and interpretability, SCARS-2 aimed to improve survival prediction and clinical decision-making for IHCA, enhancing patient outcomes.

## Methods

### Data source, collection and outcome

We used data from the Swedish Cardiopulmonary Resuscitation Registry (SCRR), which include cases of IHCA from 2008 onwards. In this study, we included data from 2010 to 2020. The SCRR collects data from all 76 Swedish hospitals with rescue teams, achieving an ascertainment level of over 95%. This registry is further enriched by merging it with the Swedish Patient Registry and the Prescribed Drug Registry. The Patient Registry includes information regarding the International Classification of Diseases (ICD) discharge codes from outpatient and inpatient clinics. We had access to ICD codes from ICD chapters A through Z. The Prescribed Drug Registry uses the Anatomical Therapeutic Chemical classification to classify medications. With regards to prescribed drugs, we had access to all drugs from sections A (alimentary tract and metabolism), B (blood and blood-forming organs) and C (cardiovascular system). The primary outcome of this study was 30-day survival following IHCA.

### Data preprocessing and feature selection

To ensure clinical applicability, the prediction model was designed for use on the arrival of the rescue team, which typically occurs within the first 15 min after collapse. As such, we limited the variables to those measured within this 15 min window. This timeframe allows for early prognostication while providing enough time for initial clinical assessment.^13^ Key variables affected by this time constraint, included, time to defibrillation and time to return of spontaneous circulation (ROSC), with any values recorded beyond 15 min or those missing classified as ‘other’. Variables collected after the 15 min window, such as those related to emergency angiography, targeted temperature management and extracorporeal membrane oxygenation, were excluded from the analysis to focus on early prognostic factors. The Cerebral Performance Category (CPC) score at hospitalisation, though important, was excluded due to its retrospective nature and unavailability in real-time clinical settings. Additionally, variables related to specific hospital units, regions or COVID-19 diagnoses were excluded for broader applicability.

From an initial set of 393 candidate predictors, we used XGBoost, LightGBM and CatBoost to select the most relevant features for survival prediction. Feature importance scores, derived using the built-in functions of these algorithms, guided the retention of the top 40 features for further analysis. This approach allowed us to balance model complexity and predictive power while ensuring that the model remained interpretable for clinical use.

### ML model development and evaluation

We evaluated several advanced ML models, including XGBoost, LightGBM and CatBoost, alongside traditional logistic regression for comparison. These models were selected for their ability to capture complex nonlinear relationships and interactions within clinical data. XGBoost effectively handles imbalanced class distributions, common in clinical data with survival and nonsurvival cases, while CatBoost processes categorical variables without extensive preprocessing. These models were selected for their proven effectiveness in clinical datasets, where handling missing data and complex feature interactions is critical.

Data preparation and processing are illustrated in online supplemental figure 1, which provides a flow chart of the pipeline, encompassing data collection, cleaning, splitting, modelling and evaluation. Stratified sampling with fivefold cross-validation was used for hyperparameter optimisation, with each fold serving as an internal validation set. Stratified splitting ensured that the distribution of key variables and survival outcomes was maintained across all subsets. Normalisation was applied only when necessary, as models like XGBoost, LightGBM and CatBoost are inherently robust to feature scale variations, while logistic regression required standard scaling for compatibility.

Hyperparameter tuning was conducted using Optuna,^14^ a Bayesian optimisation framework, to efficiently explore the hyperparameter space. To minimise the risk of overfitting, we implemented early stopping and halting training when no improvement in validation performance was observed. This approach ensures that the model generalises well to unseen data by preventing it from learning noise in the training set.

We optimised model performance using four distinct strategies: maximising AUROC to achieve high discriminatory ability, minimising calibration difference to ensure that predicted probabilities closely aligned with observed outcomes, optimising the F1-score to balance sensitivity and precision and minimising the false negative rate (FNR) to reduce the clinical risk of misclassifying patients with favourable survival prospects. After hyperparameter optimisation, the final models were retrained with the optimised parameters, improving their performance on unseen data. Details regarding the final optimisation strategy and hyperparameters for each model, including the tuning process and parameter settings, are provided in online supplemental material under the ‘Optimisation Strategy and Final Parameters’section.

We did not explicitly address class imbalance through techniques like Synthetic Minority Over-sampling Technique^15^; however, models such as CatBoost and XGBoost handle class imbalance natively. CatBoost incorporates automatic class weighting, adjusting for the unequal distribution of survival and nonsurvival cases. XGBoost is also effective in datasets with skewed class distributions, contributing to improved performance even in imbalanced scenarios. While these models, along with LightGBM, inherently manage missing data, we implemented two imputation techniques, namely, multiple imputation by chained equations (MICE) and mean imputation for comparability with logistic regression. All ML models were evaluated with and without imputation, alongside logistic regression, to assess the impact of missing data handling.

Model performance was evaluated (using fivefold cross-validation) using various metrics, including AUROC with a 95% CI, sensitivity, specificity, positive predictive value (PPV), negative predictive value (NPV), F1-score, Matthew’s correlation coefficient (MCC), false positive rate (FPR), FNR, Brier score and calibration plots. For interpretability, we employed SHAP plots to visualise the contribution of each feature to individual predictions. This method provided transparency by showing how each feature influenced predictions, enhancing clinical applicability and trust in the model’s decisions.

### Patient and public involvement

None.

## Results

Model parameters, hyperparameter optimisation and other details are provided in online supplemental materials, which also include the processing flow and optimisation methodologies.

### Baseline characteristics

A total of 25 905 patients were included in the study, with 8166 surviving for 30 days. Key baseline characteristics are summarised in table 1. The mean age was 71.7 years, with 61.8% of patients being men. Most cardiac arrests were witnessed (79.7%), and 53.2% were monitored by ECG. The history of heart failure was more common than that of acute myocardial infarction, and approximately one in four patients had a history of diabetes. Ejection fraction data were available for 17 760 individuals, with a mean value of 43%, and no significant differences were found between survivors and nonsurvivors.

**Table 1:** Characteristics of 25 905 patients with in-hospital cardiac arrest

	Overall	Alive	Dead	SMD
N	25 905	8166	17 739	
Sex, n (%)				
Men	16 000 (61.8)	5270 (64.5)	10 730 (60.5)	0.084
Women	9905 (38.2)	2896 (35.5)	7009 (39.5)	
Age, mean (SD)	71.7 (14.9)	67.2 (16.3)	73.8 (13.7)	0.436
Location of IHCA, n (%)				
Coronary Care Unit (CCU)	4015 (15.5)	1778 (21.8)	2237 (12.6)	0.678
Cath lab	2068 (8.0)	1305 (16.0)	763 (4.3)	
Emergency Room (ER)	2854 (11.0)	992 (12.1)	1862 (10.5)	
High-dependency unit	227 (0.9)	68 (0.8)	159 (0.9)	
Intensive Care Unit (ICU)	2368 (9.1)	834 (10.2)	1534 (8.6)	
Operating Room (OR)	526 (2.0)	320 (3.9)	206 (1.2)	
Other	576 (2.2)	224 (2.7)	352 (2.0)	
Paraclinical unit	1080 (4.2)	381 (4.7)	699 (3.9)	
Regular ward	12 191 (47.1)	2264 (27.7)	9927 (56.0)	
Witnessed IHCA, n (%)				
Missing	399 (1.5)	101 (1.2)	298 (1.7)	0.537
No	4856 (18.7)	494 (6.0)	4362 (24.6)	
Yes	20 650 (79.7)	7571 (92.7)	13 079 (73.7)	
ECG monitoring, n (%)				
Missing	351 (1.4)	100 (1.2)	251 (1.4)	0.608
No	11 779 (45.5)	2130 (26.1)	9649 (54.4)	
Yes	13 775 (53.2)	5936 (72.7)	7839 (44.2)	
Previous cancer, n (%)				
Missing	1687 (6.5)	491 (6.0)	1196 (6.7)	0.214
No	19 509 (75.3)	6621 (81.1)	12 888 (72.7)	
Yes	4709 (18.2)	1054 (12.9)	3655 (20.6)	
Previous diabetes, n (%)				
Missing	1302 (5.0)	441 (5.4)	861 (4.9)	0.122
No	17 948 (69.3)	5921 (72.5)	12 027 (67.8)	
Yes	6655 (25.7)	1804 (22.1)	4851 (27.3)	
Previous heart failure, n (%)				
Missing	2520 (9.7)	689 (8.4)	1831 (10.3)	0.212
No	15 411 (59.5)	5429 (66.5)	9982 (56.3)	
Yes	7974 (30.8)	2048 (25.1)	5926 (33.4)	
Previous acute MI, n (%)				
Missing	1963 (7.6)	581 (7.1)	1382 (7.8)	0.032
No	18 321 (70.7)	5847 (71.6)	12 474 (70.3)	
Yes	5621 (21.7)	1738 (21.3)	3883 (21.9)	
Ejection fraction, mean (SD)*	42.6 (14.3)	43.5 (13.8)	42.0 (14.5)	−0.104
Creatinine, mean (SD)*	146.6 (157.2)	126.5 (174.3)	155.8 (147.9)	0.181
Time to rescue team alert, median (Q1, Q3)	0.0 (0.0, 1.0)	0.0 (0.0, 1.0)	1.0 (0.0, 1.0)	0.175
Time to CPR, median (Q1, Q3)	0.0 (0.0, 1.0)	0.0 (0.0, 0.0)	0.0 (0.0, 1.0)	0.122
Time to rescue team arrival, median (Q1, Q3)	3.0 (2.0, 5.0)	3.0 (2.0, 4.0)	3.0 (2.0, 5.0)	0.177
Initial rhythm, n (%)				
Asystole	8481 (32.7)	1896 (23.2)	6585 (37.1)	0.878
Missing	6121 (23.6)	1836 (22.5)	4285 (24.2)	
PEA	5745 (22.2)	804 (9.8)	4941 (27.9)	
VF	3850 (14.9)	2566 (31.4)	1284 (7.2)	
VT	1708 (6.6)	1064 (13.0)	644 (3.6)	
Consciousness on rescue team arrival, n (%)				
Missing	3258 (12.6)	1635 (20.0)	1623 (9.1)	0.984
No	18 983 (73.3)	3716 (45.5)	15 267 (86.1)	
Yes	3664 (14.1)	2815 (34.5)	849 (4.8)	
Pulse on rescue team arrival, n (%)				
Missing	4761 (18.4)	2081 (25.5)	2680 (15.1)	0.977
No	15 559 (60.1)	2534 (31.0)	13 025 (73.4)	
Yes	5585 (21.6)	3551 (43.5)	2034 (11.5)	
IHCA preceded by arrhythmia, n (%)				
Missing	16 890 (65.2)	4107 (50.3)	12 783 (72.1)	0.527
No	3656 (14.1)	1185 (14.5)	2471 (13.9)	
Yes	5359 (20.7)	2874 (35.2)	2485 (14.0)	
Ongoing acute Myocardial Infarction (MI), n (%)				
Missing	2963 (11.4)	579 (7.1)	2384 (13.4)	0.269
No	17 052 (65.8)	5243 (64.2)	11 809 (66.6)	
Yes	5890 (22.7)	2344 (28.7)	3546 (20.0)	
Epinephrine administered, n (%)				
Missing	791 (3.1)	348 (4.3)	443 (2.5)	1.159
No	8584 (33.1)	5383 (65.9)	3201 (18.0)	
Yes	16 530 (63.8)	2435 (29.8)	14 095 (79.5)	
Antiarrhythmics administered, n (%)				
Missing	1795 (6.9)	502 (6.1)	1293 (7.3)	0.184
No	20 485 (79.1)	6157 (75.4)	14 328 (80.8)	
Yes	3625 (14.0)	1507 (18.5)	2118 (11.9)	

* Values in the previous 6 months, if available.

IHCA, in-hospital cardiac arrest; PEA, pulseless electrical activity; SMD, Standardised Mean Difference; VF, ventricular fibrillation; VT, ventricular tachycardia.

Other key variables included time to alert the rescue team (median 0 min), time to rescue team arrival (median 3 min) and initial rhythm. Initial rhythm data were missing in 23.6% of patients; among those with available data, 32.7% had asystole, 22.2% had pulseless electrical activity, 6.6% had ventricular tachycardia and 14.9% had ventricular fibrillation.

### Impact of imputation on model performance and statistical significance

To address missing data, we performed Little’s MCAR test, revealing that the missing data are more likely missing at random or missing not at random (MNAR). Specifically, pulse on arrival (65.2% missing) was MNAR, depending on whether emergency responders could measure it, while ejection fraction (31.4% missing) was often unavailable due to the lack of echocardiography. Table 2 presents the performance of CatBoost, XGBoost, LightGBM and logistic regression, comparing results with and without imputation. The table highlights the impact of missing data on key metrics such as AUROC, Brier score, sensitivity, specificity and F1-score. CatBoost demonstrated stable performance across both imputation methods, with minimal variation, while XGBoost and LightGBM showed slight decreases in sensitivity and specificity with MICE imputation. Logistic regression underperformed in sensitivity (0.6985 vs 0.7117 for CatBoost), specificity (0.9157 vs 0.9287 for CatBoost) and F1-score (0.7425 vs 0.7626 for CatBoost), with a higher Brier score (0.1122 vs 0.1028 for CatBoost). The calibration plots for all models, along with the Brier score (using mean imputation for comparability), are presented in online supplemental figure 2.

**Table 2:** Performance evaluation of CatBoost, XGBoost, LightGBM and logistic regression models with and without imputation using fivefold cross-validation

With all features (fivefold cross-validation)			
	Without imputation	With imputation	
		Mean	MICE
CatBoost			
AUROC	0.9136	0.9135	0.9133
95% CI	(0.9075 to 0.9191)	(0.9075 to 0.9185)	(0.9082 to 0.9185)
Brier score	0.1028	0.1029	0.1031
PPV	0.8214	0.8196	0.8185
Sensitivity	0.7117	0.7088	0.7116
Specificity	0.9287	0.9281	0.9273
Mean F1-score	0.7626	0.7601	0.7613
XGBoost			
AUROC	0.9123	0.9100	0.9086
95% CI	(0.9057 to 0.9183)	(0.9015 to 0.9166)	(0.9017 to 0.9137)
Brier score	0.1035	0.1052	0.1064
PPV	0.8250	0.8140	0.8231
Sensitivity	0.7046	0.7121	0.6905
Specificity	0.9311	0.9250	0.9313
F1-score	0.7600	0.7594	0.7505
LightGBM			
AUROC	0.9133	0.9125	0.9085
95% CI	(0.9071 to 0.9194)	(0.9062 to 0.9192)	(0.9010 to 0.9142)
Brier score	0.1027	0.1035	0.1061
PPV	0.8219	0.8153	0.8138
Sensitivity	0.7137	0.7165	0.6986
Specificity	0.9287	0.9250	0.9262
F1-score	0.7639	0.7625	0.7514
Logistic regressions			
AUROC	–	0.8976	0.8976
95% CI	–	(0.8913 to 0.9038)	(0.8913 to 0.9038)
Brier score	–	0.1122	0.1122
PPV	–	0.7924	0.7920
Sensitivity	–	0.6985	0.6979
Specificity	–	0.9157	0.9156
F1-score	–	0.7425	0.7420

AUROC, area under the receiver operating characteristics; MICE, multiple imputation by chained equations; PPV, positive predictive value.

To statistically validate the superior performance of CatBoost and other ML models (XGBoost and LightGBM) over logistic regression, we conducted multiple statistical tests. Paired t-tests confirmed that all ML models significantly outperformed logistic regression in terms of AUROC, with CatBoost showing the most substantial difference (t=17.428, p=0.000), followed by LightGBM (t=11.813, p=0.000) and XGBoost (t=4.433, p=0.011). Additionally, decision curve analysis (online supplemental figure 3) demonstrated that the ML models consistently provided a superior net clinical benefit compared with logistic regression, highlighting their enhanced decision-making capability in real-world clinical settings. The Hosmer-Lemeshow test was performed for logistic regression and confirmed that the model exhibited good calibration and fit, with most folds showing a satisfactory fit.

### Feature importance and optimisation strategy

Given CatBoost’s strong performance in handling missing data (table 2), we conducted a two-step feature selection process to identify key survival predictors. First, we applied feature importance analysis using default parameters to narrow down the initial 393 features to the top 40 (online supplemental figure 4). For comparison, feature selection results for XGBoost and LightGBM are also presented in the same figure. This step ensured that only the most relevant variables contributing to model performance were retained while maintaining interpretability and efficiency. Next, we further refined the feature set using different optimisation strategies. Since minimising false negatives (FNR) is particularly critical in clinical decision-making, we prioritised an FNR-optimised model for the final reduced feature set. Notably, feature importance rankings remained stable across different optimisation approaches, reinforcing the robustness of selected predictors. Online supplemental figure 5a highlights the top 20 predictors identified by the FNR-optimised CatBoost model, with epinephrine administration, age, initial rhythm and ROSC as particularly significant predictors emerging within 15 min.

Using the reduced feature set, CatBoost achieved peak performance with fewer than 10 predictors. Online supplemental figure 5b illustrates AUROC performance as the top 15 predictors were added sequentially. AUROC steadily improved as more predictors were incorporated, stabilising after the addition of eight features and reaching 0.9024, surpassing XGBoost (0.8538) and LightGBM (0.8660). This analysis underscores the benefits of eliminating redundant features to enhance model efficiency while preserving predictive accuracy.

### CatBoost performance with top 15 predictors

**Figure 1:**
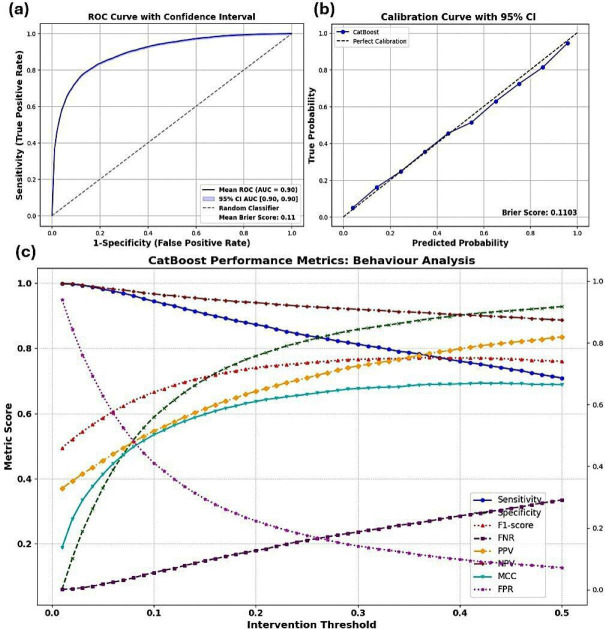
Performance evaluation of the FNR-optimised CatBoost model with top 15 predictors (evaluated with fivefold cross-validation). (**a**) AUROC curve demonstrated the model’s discrimination ability. (**b**) Calibration plot illustrating strong agreement between predicted probabilities and actual outcomes, with a Brier score of 0.11 and 95% CIs. (**c**) Threshold analysis displaying the behaviour of key metrics (eg, sensitivity, specificity, F1-score, FNR, PPV, NPV, MCC and FPR) across thresholds from 1% to 50%, offering insights into clinical trade-offs between sensitivity and specificity. AUROC, area under the receiver operating characteristics; FNR, false negative rate; FPR, false positive rate; MCC, Matthew’s correlation coefficient; NPV, negative predictive value; PPV, positive predictive value.

Figure 1 illustrates CatBoost’s performance, using the top 15 most influential features identified by CatBoost’s feature importance evaluation. The AUROC was 0.9034 (95% CI 0.8955 to 0.9037), with a Brier score of 0.1103, reflecting good predictive accuracy. Figure 1a shows the AUROC, providing a visual representation of the model’s ability to discriminate between survival and nonsurvival outcomes. Figure 1b presents the calibration plot, demonstrating strong alignment between the predicted probabilities and actual outcomes, with a 95% CI. This indicated minimal underestimation of survival likelihood, which was crucial for clinical decision-making.

Figure 1c illustrates the performance of key across thresholds from 1% to 50%. Sensitivity remained high at lower thresholds and gradually decreased, reaching 0.70 at 50%, indicating strong true positive detection at lower thresholds. Specificity increased with stricter thresholds, reaching 0.92 at 45%, reflecting improved identification of nonsurvivors. The F1-score peaked at 0.75 at 15%, while FNR increased from 0.002 at 1% to 0.30 at 45%, reflecting the trade-off between sensitivity and false negatives. PPV improved with higher thresholds, rising from 0.34 at 1% to 0.80 at 45%. NPV remained high, starting at 1.00 and decreasing to 0.87 at 45%, indicating consistent true negative detection. MCC improved, peaking at 0.64 at 45%, suggesting a better overall balance. FPR decreased from 0.91 at 1% to 0.08 to 45%, indicating fewer false positives at stricter thresholds.

### Explainability and feature impact

**Figure 2:**
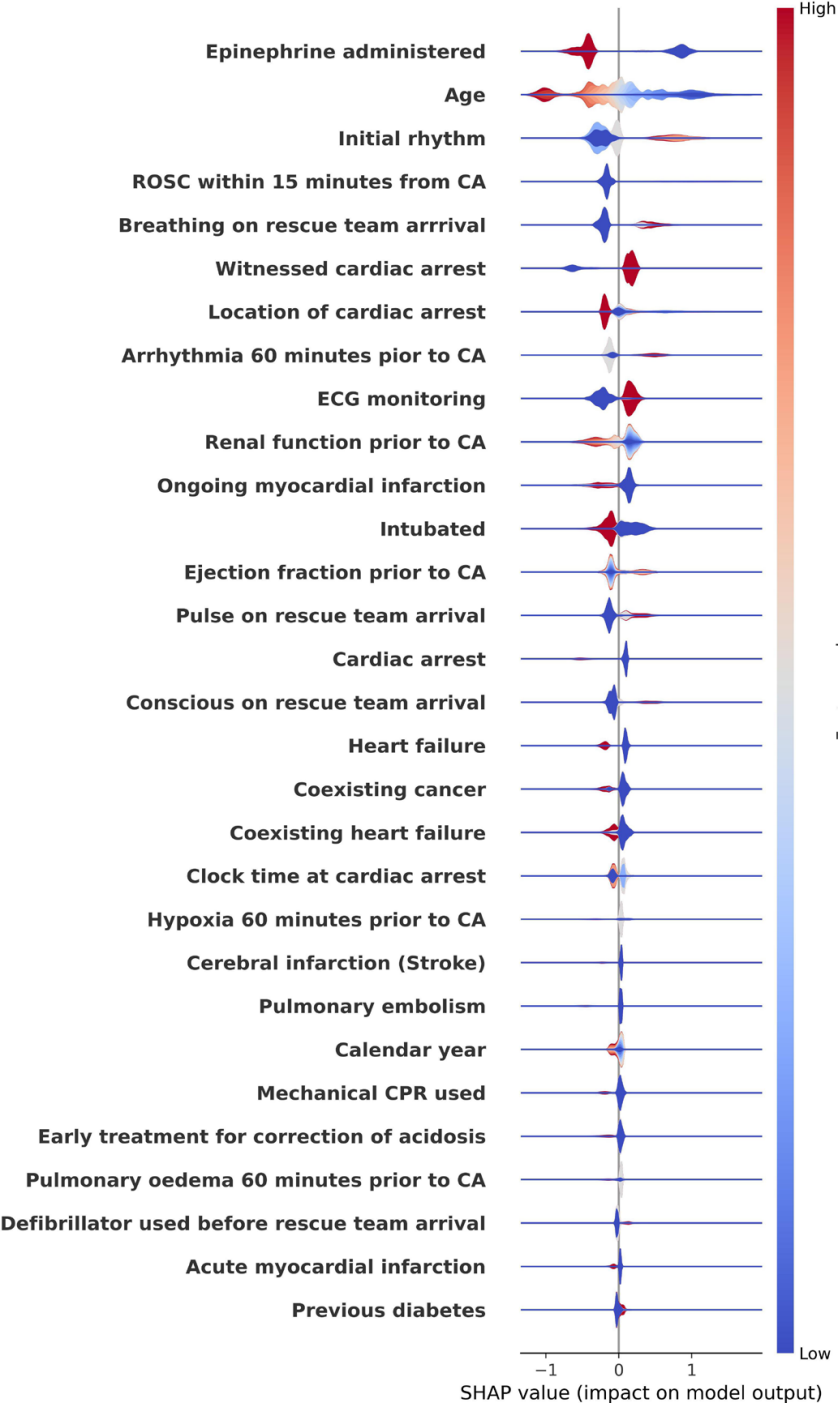
SHAP violin plot of feature impact on survival predictions. The violin plot ranks features by their mean absolute SHAP values, illustrating their importance to the model’s predictions on testing data. Each point represents a patient case, with SHAP values along the x-axis showing the feature’s impact. Epinephrine administration demonstrates a strong negative effect on survival, while age shows variable influence. The colour gradient (red for high values and blue for low values) highlights feature variability across cases. CA, cardiac arrest; CPR, cardiopulmonary resuscitation; ROSC, return of spontaneous circulation; SHAP, Shapley Additive Explanations.

**Figure 3:**
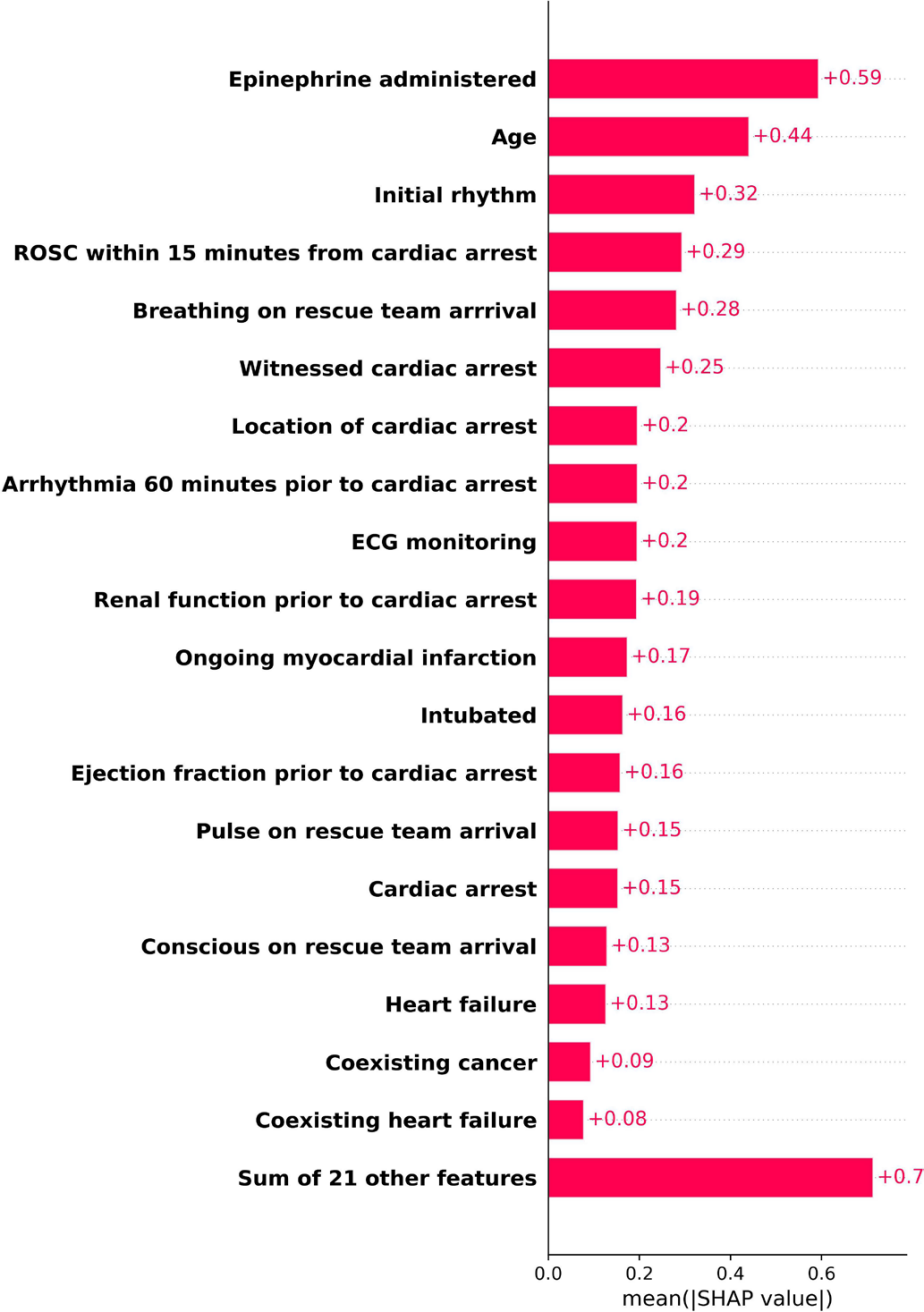
SHAP bar plot of feature importance for 30-day survival prediction. This bar plot displays the most influential predictors of survival, ranked by their mean absolute SHAP values on testing data. Features such as epinephrine administration, age, initial rhythm, ROSC within 15 min and breathing on rescue team arrival show varying levels of impact on survival outcomes. ROSC, return of spontaneous circulation; SHAP, Shapley Additive Explanations.

To gain insights into model decision-making, we used SHAP analysis to evaluate feature contributions. Figure 2 presents a violin plot and figure 3 a bar plot of SHAP values, highlighting the most influential predictors of 30-day survival. Epinephrine administration (SHAP value: 0.59) and age (0.44) were the most significant, with higher epinephrine levels and increasing age correlating with poorer survival outcomes. Other important features included initial rhythm, ROSC within 15 min and breathing on rescue team arrival, all indicative of survival likelihood.

SHAP analysis provided deeper insights into how these features influence individual predictions. The violin plot in figure 2 showed that epinephrine administration had a strong negative effect on survival, while the age spread was broader, reflecting a varying impact on survival outcomes. These findings confirm the clinical relevance of the identified features and support their role in guiding medical decision-making. Online supplemental figure 6 shows the decision plot, providing a visual representation of how individual features affect the model’s predictions for individual cases, offering further interpretability of the model’s decision-making process.

### Clinical application of the model

The final model, SCARS-2, based on CatBoost, optimised for FNR, has been implemented as an online API tool for clinical use. Its robust performance and high interpretability make it a valuable resource for clinicians in predicting 30-day survival, offering a practical solution for real-time decision-making in emergency and critical care settings.

## Discussion

In this study, we developed SCARS-2, a prediction model for 30-day survival after IHCA, using a dataset of 26 000 cases. SCARS-2 was designed for real-time clinical use, providing survival predictions within 15 min of collapse using readily available clinical variables such as age, epinephrine administration, initial rhythm and ROSC. The model (CatBoost) demonstrated excellent performance, with an AUROC of 0.9136 (95% CI 0.9075 to 0.9191) and a Brier score of 0.1028, outperforming other ML frameworks such as XGBoost, LightGBM and logistic regression.

When using CatBoost with the top 15 features selected, it achieved an area under the curve (AUC) of 0.9034 (95% CI 0.8955 to 0.9037) and a Brier score of 0.1103. In comparison, XGBoost and LightGBM demonstrated lower performance, with AUCs of 0.8538 (95% CI 0.8483 to 0.8585) and 0.8678 (95% CI 0.8571 to 0.8810), and Brier scores of 0.1539 and 0.1250, respectively.

The superiority of CatBoost-selected features was further demonstrated when these features were used in a logistic regression model. Logistic regression, trained with the top 15 features selected by each ML model, yielded the following results: with CatBoost-selected features, the mean AUC was 0.8678 (95% CI 0.8571 to 0.8810) and the mean Brier score was 0.1250. In contrast, using XGBoost-selected features, the mean AUC was 0.8218 (95% CI 0.8122 to 0.8269) with a mean Brier score of 0.1476. With LightGBM-selected features, the model achieved a mean AUC of 0.8348 (95% CI 0.8203 to 0.8537) and a Brier score of 0.1414. These findings highlight the superior feature selection performance of CatBoost, which consistently outperformed both XGBoost and LightGBM when used in conjunction with logistic regression.

In this study, we leveraged the in-built feature importance evaluation of CatBoost, offering key advantages over traditional methods like Least Absolute Shrinkage and Selection Operator (LASSO) or Recursive Feature Elimination. CatBoost simplifies model development by handling complex datasets without the need for extensive manual feature selection. Its proven effectiveness in handling large datasets with missing values and maintaining predictive accuracy makes it especially suitable for clinical settings. In experiments comparing imputed with non-imputed data, we found that CatBoost consistently outperformed other methods, demonstrating robust performance regardless of imputation. Unlike traditional models that rely on imputation techniques, CatBoost natively handles missing data, ensuring robustness in real-world clinical scenarios, such as sudden cardiac arrest, where key variables like pulse or initial rhythm are often unavailable. Additionally, CatBoost addresses class imbalance, which is critical for predicting the rare survival outcomes in IHCA, further enhancing the model’s predictive accuracy and clinical applicability. In addition to CatBoost’s strong performance, it can be tailored to meet specific clinical needs, as it was optimised to minimise the FNR, ensuring better identification of patients with survival potential.

SCARS-2 performed well across different decision thresholds, showing excellent sensitivity at low thresholds which gradually decreased to 0.70 at 50%, while specificity improved significantly (0.92 at 45%). The F1-score peaked at 0.75 at 15%, indicating a good balance between sensitivity and precision. PPV showed notable improvement from 0.34 to 0.0.80, and NPV remained high, ensuring reliable detection of true negatives. However, FNR increased with stricter thresholds, highlighting the trade-off between sensitivity and specificity. These results underscore SCARS-2’s flexibility, allowing for fine-tuning based on clinical priorities while maintaining high diagnostic performance.

The clinical utility of SCARS-2 extends beyond its predictive accuracy to its interpretability. CatBoost’s built-in feature importance evaluation and SHAP analysis provide insights into the most influential variables driving the model’s predictions. Key predictors, such as epinephrine administration, age, initial rhythm and ROSC, align closely with established clinical knowledge, further supporting the model’s real-world applicability. For example, ROSC within 15 min reflects the effectiveness of early resuscitation efforts, while epinephrine administration is a cornerstone of advanced cardiac life support protocols. Additional predictors like breathing and consciousness status on rescue team arrival, arrhythmia prior to cardiac arrest and renal function further highlight the importance of capturing both immediate clinical observations and underlying patient physiology.

Compared with earlier cardiac arrest prediction models, SCARS-2 stands out in terms of its simplicity, efficiency and clinical applicability. Many earlier models^16 17^ often required complex feature engineering and extensive preprocessing, which could delay real-time predictions and hinder clinical deployment. Unlike other models such as GO-FAR, National Early Warning Score (NEWS) and ICU mortality scores, SCARS-2 was specifically developed for predicting 30-day survival following IHCA in general hospital settings, particularly after 15 min of resuscitation. GO-FAR, for instance, is designed to predict the likelihood of survival with a good neurological outcome but is typically applied at the time of hospital admission for adult patients, making it more suitable for early-stage assessments. NEWS, on the contrary, is used to monitor the clinical deterioration of adult patients and is focused on identifying those who are at risk of adverse events, rather than predicting survival postcardiac arrest. ICU-specific models like Simplified Acute Physiology Score II (SAPS II) or Acute Physiology And Chronic Health Evaluation (APACHE) predict mortality within the ICU, focusing on critically ill patients who are already admitted to intensive care, a population that represents only a small fraction of cardiac arrest survivors. In contrast, SCARS-2 is tailored to evaluate survival chances after a cardiac arrest event in any ward setting, where patients may be in various states of recovery following resuscitation. Its focus on the post-resuscitation period (around 15 min after collapse) allows for real-time clinical decision-making, directly addressing the needs of physicians who must decide on the potential outcomes for patients in the critical postarrest phase. The distinct focus and patient population make direct comparisons to other risk scores less relevant.

Recognising the differences in application and patient populations, SCARS-2 is made publicly available via GitHub (https://github.com/Vibha190685/Predicting-30-day-survival-after-in-hospital-cardiac-arrest/tree/main) to allow for broader evaluation and use across diverse healthcare settings. By offering the model to the scientific and clinical communities, we encourage its comparison with other predictive tools, fostering a better understanding of its capabilities and limitations in different clinical environments. This approach also ensures transparency and facilitates further refinement of the model based on real-world applications and feedback.

Despite its strengths, SCARS-2 has limitations that require further investigation. The model was validated using a large internal dataset, but external validation across multiple healthcare settings and patient populations is necessary to assess its generalisability. Additionally, SCARS-2 is not intended to replace clinical judgement but to complement it. While SCARS-2 offers valuable survival probability estimates, clinical expertise remains essential for interpreting these predictions in the context of individual patient circumstances.

In conclusion, SCARS-2 is an efficient, interpretable and clinically applicable tool for predicting 30-day survival following IHCA. With continued external validation and refinement, particularly in the handling of data imputation and class imbalance, SCARS-2 has the potential to improve decision-making in critical care settings and enhance patient survival outcomes.

## Supplementary material

10.1136/bmjopen-2024-090493 online supplemental file 1

## Data Availability

Data are available upon reasonable request.
