# Supplementary material for: Predicting 30-day survival after in-hospital cardiac arrest: a nationwide cohort study using machine learning and SHAP analysis
Source: BMJ Open. 2025 Apr 27;15(4):e090493. doi: 10.1136/bmjopen-2024-090493 (PMC12035429; doi:10.1136/bmjopen-2024-090493)
Supplement: online supplemental file 1 [file bmjopen-15-4-s001.docx]

**Supplementary Material**

**Title:**

**Predicting 30-day survival after in-hospital cardiac arrest: a nationwide cohort study using machine learning and SHAP analysis**

Overview:

This supplementary material supports the main manuscript by providing:

- The TRIPOD+AI checklist with page/line references.
- Additional figures key aspects of the study, including calibration plots and predictor rankings.
- Relevant data details for transparency and reproducibility.

**Section A: TRIPOD+AI Checklist**

| Item | Description | Reported (Y/N) | Page/Line |
| --- | --- | --- | --- |
| Title & Abstract | Title specifies the study type and abstract is structured. | Yes | Page 1-4 |
| Introduction | Explains the study's purpose and background clearly. | Yes | Page 5-7, Lines 1–61. |
| Study Design | Design clearly described (e.g., retrospective cohort study). | Yes | Page 7-11, Lines 64-155. |
| Participants | Inclusion/exclusion criteria and data sources detailed. | Yes | Page 7, Lines 69-71 |
| Predictors | Detailed list of predictors used in the model. | Yes | Page 18,21, Lines 259-263,332-339. Supple Figures: 4-5 |
| Missing Data | How missing data were handled. | Yes | Page 10, Lines 143-147 |
| Model Development | Description of algorithms, validation, and tuning methods. | Yes | Page 9-11, Lines 103-155. |
| Model Performance | Reported metrics (AUROC, calibration, FNR, etc.). | Yes | Page 14-18, Lines 181-254 |
| Interpretability (AI-specific) | Use of SHAP/feature importance to explain predictions. | Yes | Page 18, Lines 256–272 |
| Discussion | Strengths, limitations, and clinical implications detailed. | Yes | Page 19-23, Lines 280-381 |
| Funding & Conflicts of Interest | Funding sources and disclosures mentioned. | Yes | Page 23, Lines 383-390 |

**Section B: Figures**

**
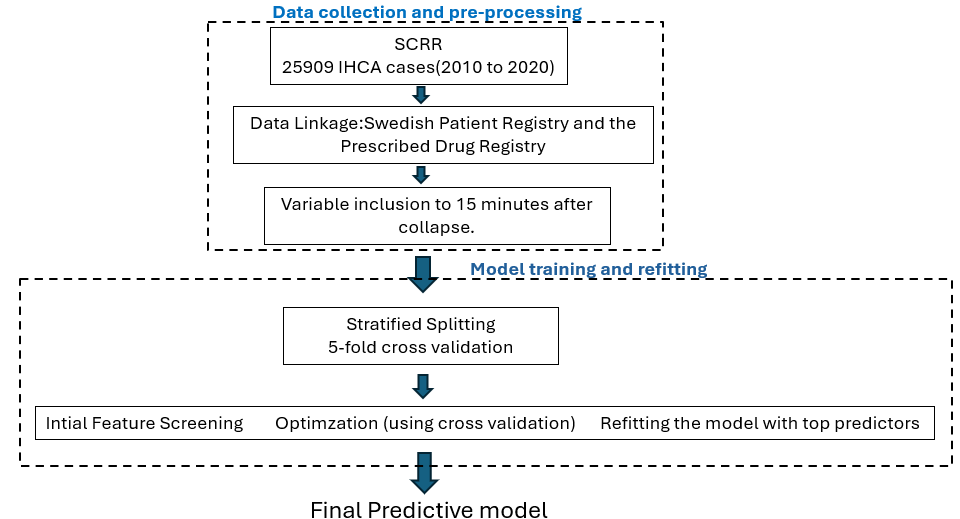
**

Supplementary Figure 1: Preprocessing Pipeline. This figure illustrates the complete workflow, including data collection, cleaning, and subsequent model training. The pipeline outlines the steps for preparing the clinical data for machine learning model development.

**
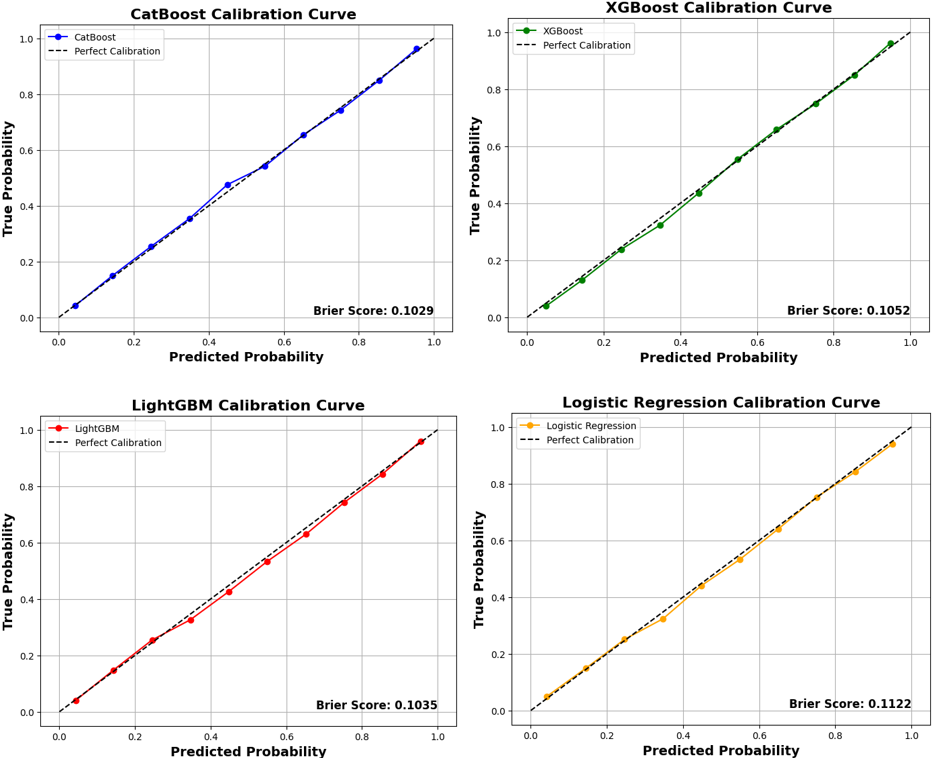
**

Supplementary Figure 2: Calibration plots for CatBoost, XGBoost, LightGBM, and logistic regression using all 393 features. All plots are computed using mean imputation and evaluated through 5-fold cross-validation. Brier Scores are provided to assess overall calibration performance.


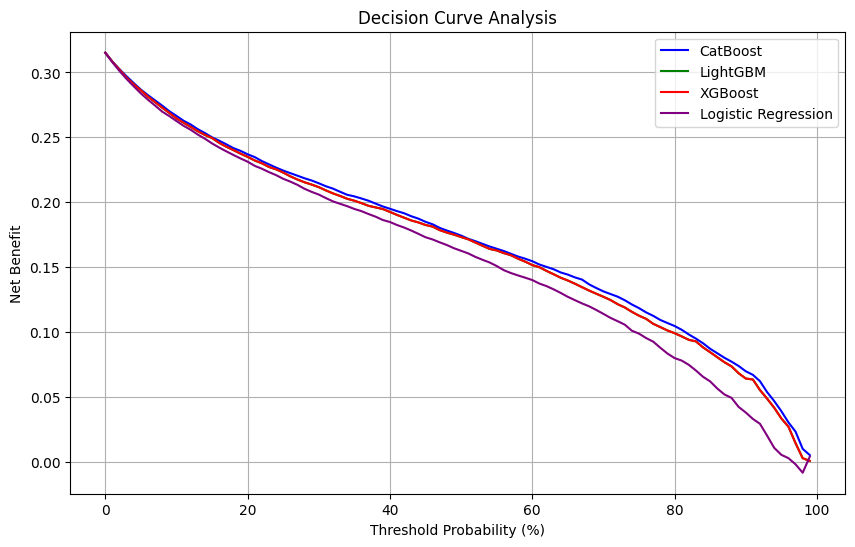


Supplementary Figure 3: Decision Curve Analysis (DCA) to Assess Net Clinical Benefit.

**(a)**


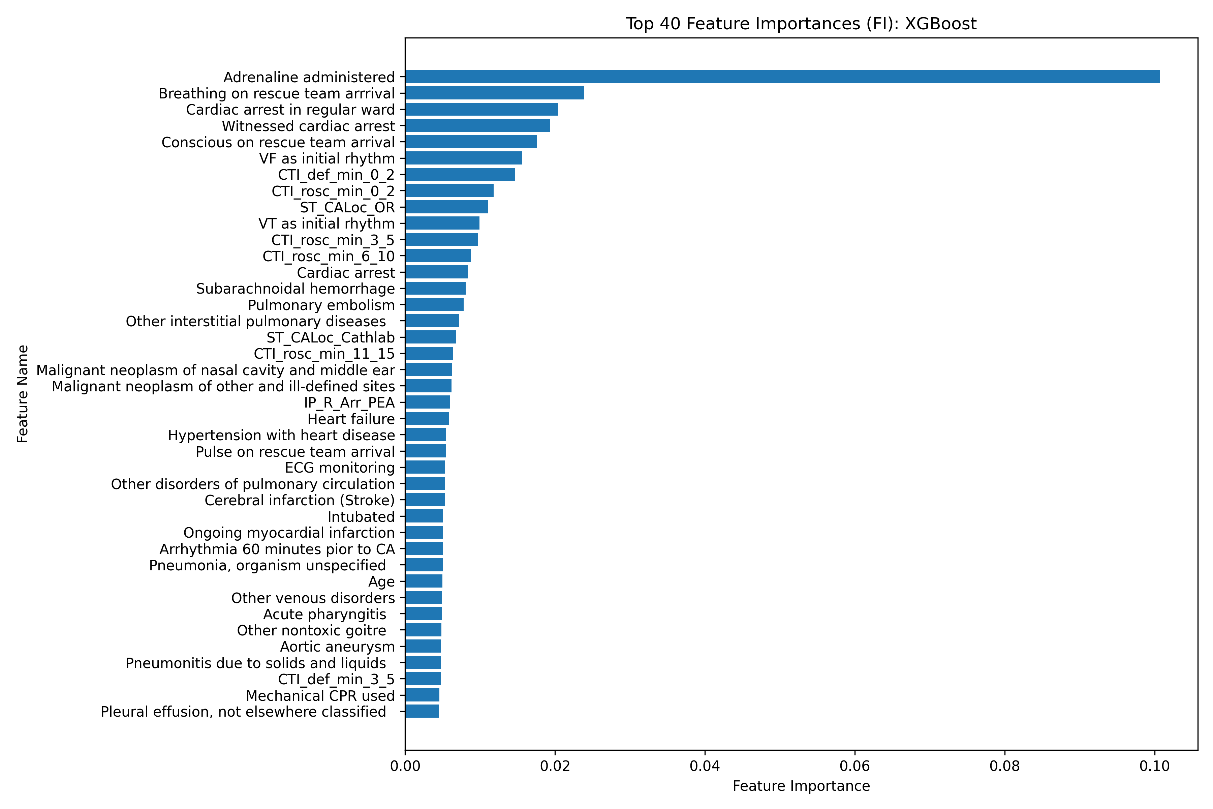


**(b)**


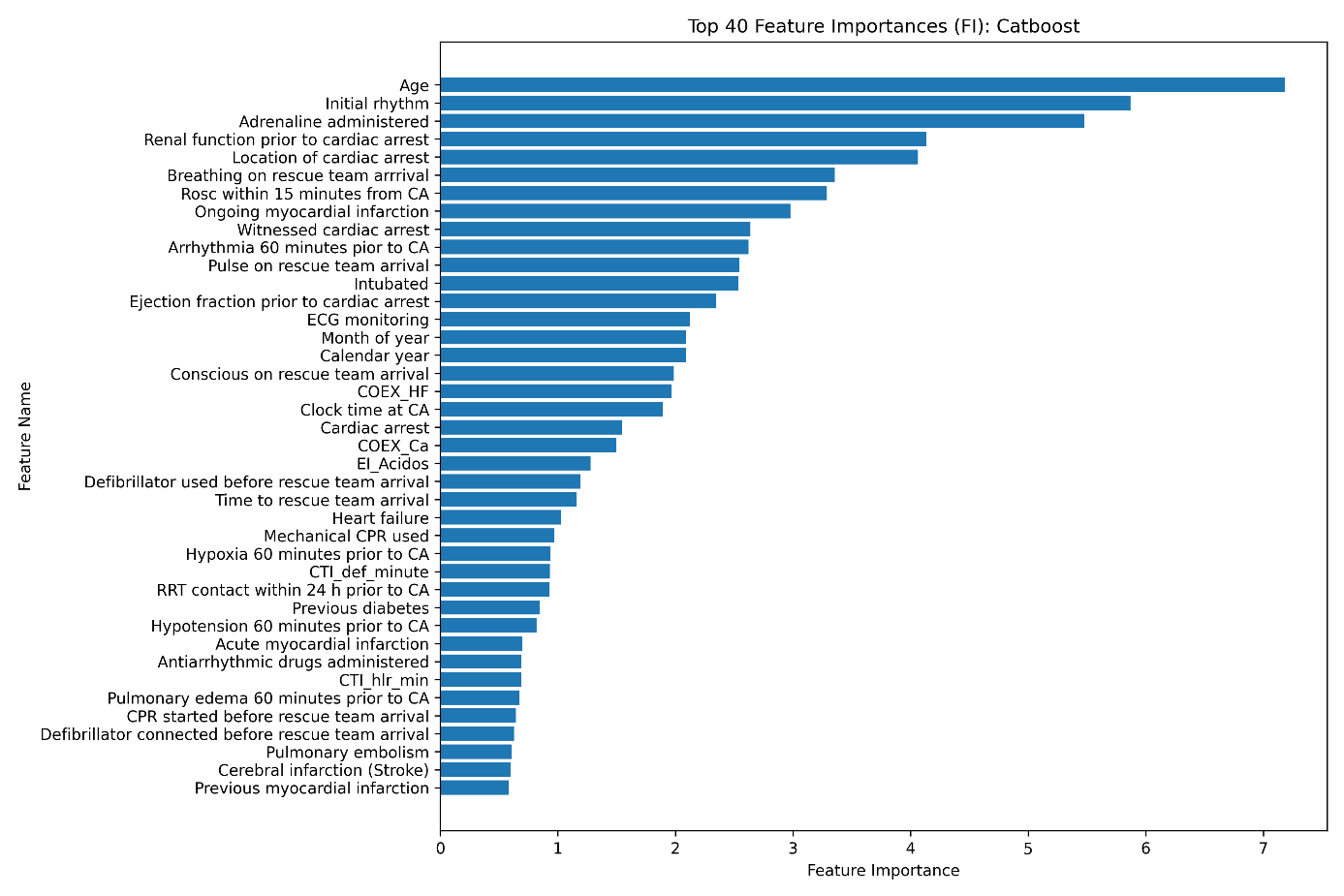


**(c)**


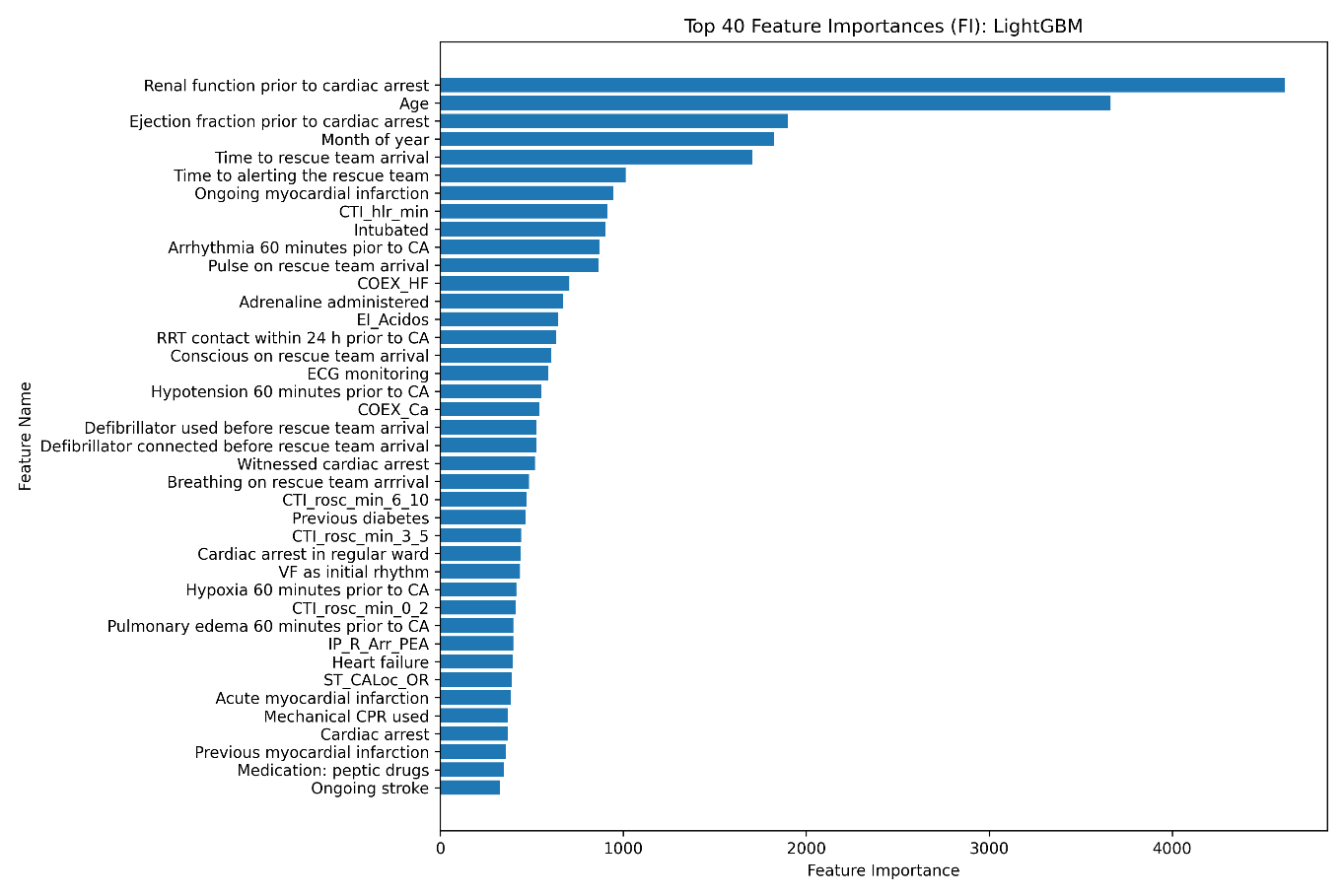


Supplementary Figure 4: Initial Feature Screening: This figure presents the ranking of the top 40 features selected from an initial set of 393 predictors. Feature importance scores were computed using (a) XGBoost, (b) LightGBM, (c) CatBoost, and in-built feature importance. While the rankings of the features varied slightly across the models, key predictors such as adrenaline administration, initial rhythm, age, ejection fraction, and critical time intervals consistently emerged as important for survival prediction.

**(a)**


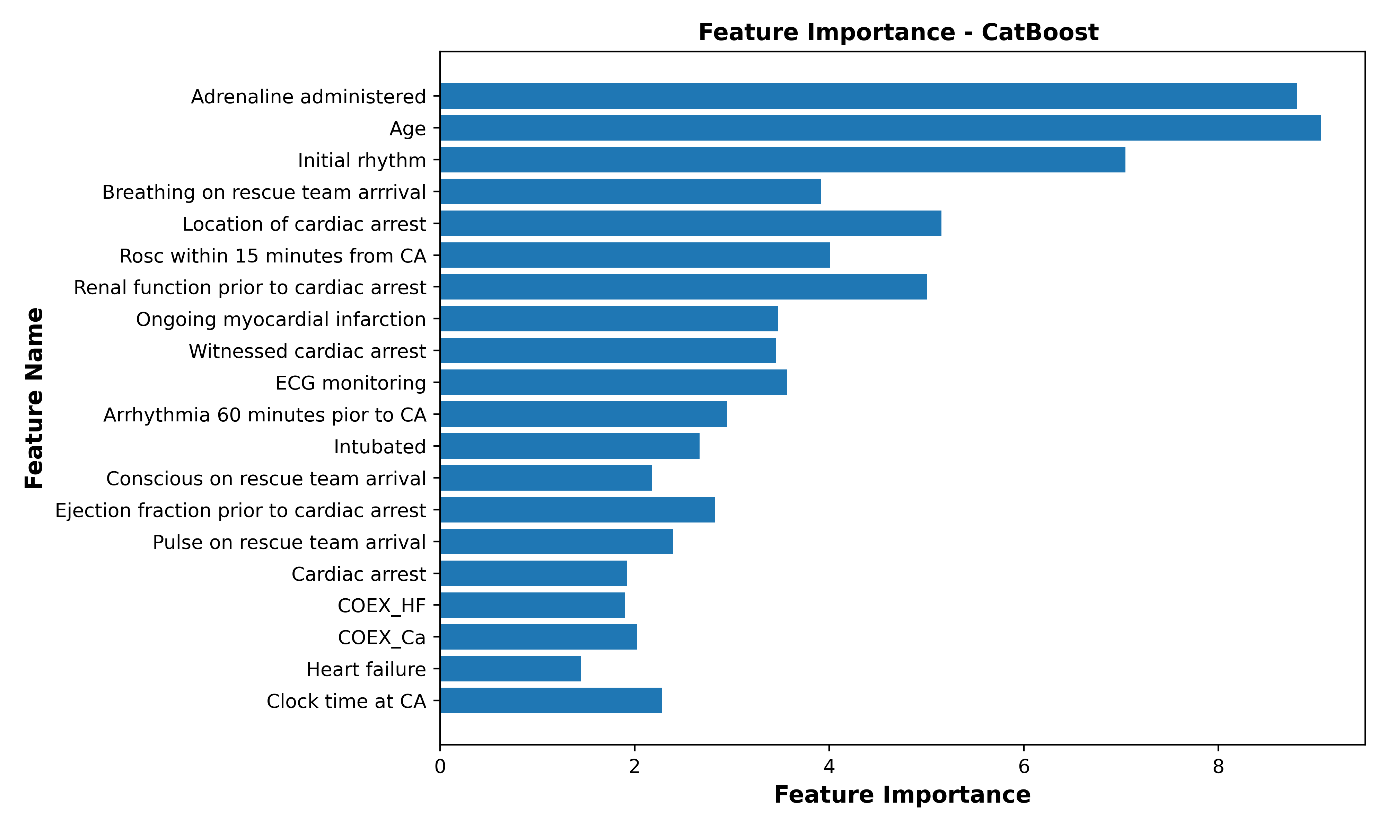


**(b)**


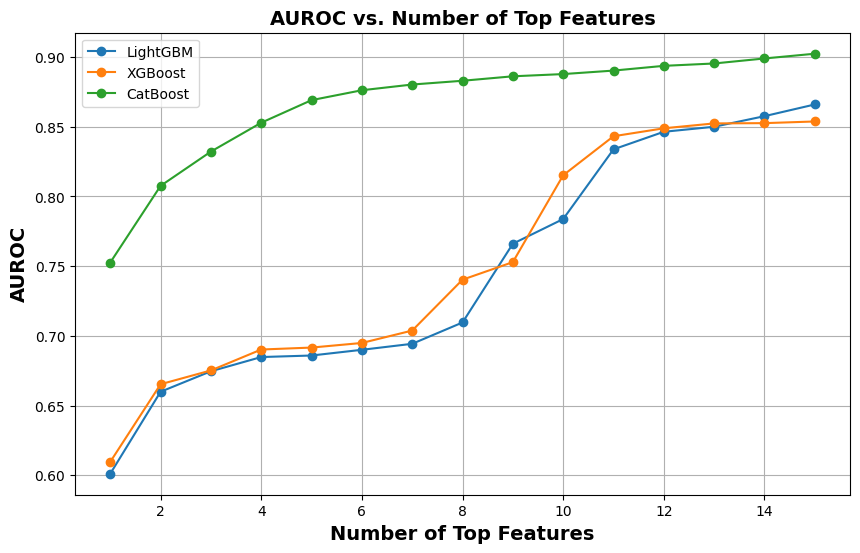


Supplementary Figure 5: Feature Importance and AUROC (a) Top 20 predictors ranked by importance in the FNR-optimized CatBoost model (optimization) including key clinical factors such as adrenaline administration and ROSC. (b) AUROC performance comparison for CatBoost, XGBoost, and LightGBM Performance (5-fold cross validation) showing the model's efficiency as predictors were sequentially added.


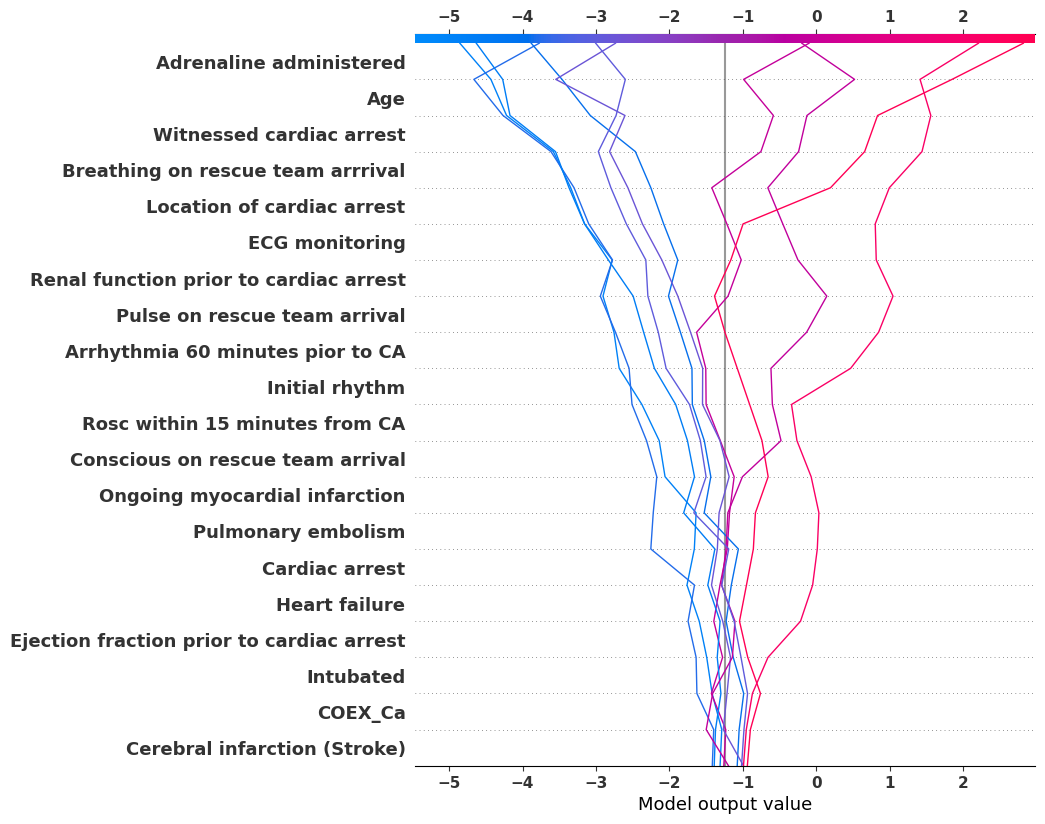


Supplementary Figure 6: Decision plot showing how individual features impact the model's predictions for specific cases, providing further interpretability into the decision-making process of the model.

**Section C: Optimization and Hosmer-Lemeshow Test details**

**Optimization Strategy and Final Parameters:**

To initialize the feature selection for the models, we began with a base configuration of 2000 trees, a learning rate of 0.01, and a maximum depth of 9. After obtaining the initial features, we optimized the model using various strategies within this range by employing five-fold cross-validation. The hyperparameters were fine-tuned using the following search space:

Iterations: trial.suggest_int('iterations', 200, 2500)

Depth: trial.suggest_int('depth', 5, 15)

Learning Rate: trial.suggest_float('learning_rate', 0.005, 0.05)

Subsample: trial.suggest_float('subsample', 0.5, 1)

Early stopping was applied when there was no improvement in the model performance during optimization.

Below are the optimized hyperparameters for the model based on different evaluation criteria:

AUCROC: {'iterations': 816, 'depth': 7, 'learning_rate': 0.045342726863657805, 'subsample': 0.8530051969650675}

Calibration: {'iterations': 1606, 'depth': 6, 'learning_rate': 0.021586963225915422, 'subsample': 0.9093159429178072}

F1-score: {'iterations': 757, 'depth': 7, 'learning_rate': 0.04893129817980298, 'subsample': 0.9848479780334152}

FNR: {'iterations': 442, 'depth': 7, 'learning_rate': 0.04842088634783629, 'subsample': 0.7856889871063337}

**Hosmer-Lemeshow Test:**

Fold 1: Hosmer-Lemeshow Test Statistic: 14.7265, p-value: 0.0647 (Model fits well)

Fold 2: Hosmer-Lemeshow Test Statistic: 5.4842, p-value: 0.7048 (Model fits well)

Fold 3: Hosmer-Lemeshow Test Statistic: 13.3835, p-value: 0.0993 (Model fits well)

Fold 4: Hosmer-Lemeshow Test Statistic: 20.1252, p-value: 0.0299 (Model does not fit well)

Fold 5: Hosmer-Lemeshow Test Statistic: 13.7108, p-value: 0.0896 (Model fits well)

From the Hosmer-Lemeshow test results, we can observe that for most folds, the logistic regression model fits the data well (p-value ≥ 0.05). However, for Fold 4, the p-value is less than 0.05, indicating that the model does not fit well for that fold.
