# Supplementary material for: Predicting 30-day survival after in-hospital cardiac arrest: a nationwide cohort study using machine learning and SHAP analysis
Source: BMJ Open. 2025 Apr 27;15(4):e090493. doi: 10.1136/bmjopen-2024-090493 (PMC12035429; doi:10.1136/bmjopen-2024-090493)
Supplement: Supplementary file 2 [file bmjopen-2024-090493.reviewer_comments.pdf]

PEER REVIEW HISTORY

BMJ Open publishes all reviews undertaken for accepted manuscripts. Reviewers are asked to complete a checklist review form and are provided with free text boxes to elaborate on their assessment. These free text comments are reproduced below.

ARTICLE DETAILS

Title (Provisional)

Predicting 30-day survival after in-hospital cardiac arrest: a nationwide cohort study using machine learning and SHAP analysis

Authors

Gupta, Vibha; Rawshani, Aidin; Lundgren, Peter; Börjesson, Mats; Råmunddal, Truls; Nordberg, Per; Djärv, Therese; Herlitz, Johan; sundström, joakim; Magnusson, Carl; Louca, Antros; Andersson, Erik; Skoglund, Kristofer; Rawshani, Araz

---

VERSION 1 - REVIEW

**Reviewer**	**1**
**Name**	**Komukai, Sho**
**Affiliation**	**Osaka University**
**Date**	**09-Sep-2024**
**COI**	**no conflict of interest**

Referee report for bmjopen-2024-090493

"Prediction of survival Outcome in-hospital cardiac arrest: The SCARS-2 Model."

The authors developed a 30-day survival prediction model for an in-hospital cardiac arrest population using machine learning techniques, focusing on minimizing the false negative rate, and evaluated its performance. They named the proposed model the SCARS-2 Model, which includes the following variables as predictors: adrenaline administration, age, initial rhythm, breathing status when the rescue team arrives, the location of the cardiac arrest, whether there was a return of spontaneous circulation (ROSC), renal function before the arrest, ongoing heart attack, whether the arrest was witnessed, ECG monitoring results, any arrhythmias before the arrest, the level of consciousness when the rescue team arrives, and ejection fraction. This article is very well discussed, but there are some comments to improve the quality of the manuscript.

Comment 1:

It appears that cases where ROSC occurred within 15 minutes are included in the analysis population. However, considering the purpose of this prediction model, I feel it would be

more useful to exclude such cases and build a prediction model for the population where ROSC did not occur within 15 minutes.

Comment 2: Regarding Missing Data

Even in clinical settings, future data collection for diagnosis should assume that measurements can be made without missing data. Considering this, a more detailed discussion is needed on whether missing data holds any significance in the prediction context.

It should also be clarified in the paper how missing values were handled within the model. Furthermore, if it is assumed that the missing data is "Missing at Random," methods such as data imputation or inverse probability weighting for missing data should be applied.

In particular, the missing rate for the initial rhythm is 26.3%, which is extremely concerning. Careful consideration is needed to determine whether the missing data is meaningful or if it is random. If it is the former, there is a possibility that bias exists in the data, which raises questions about the generalizability of the results.

Comment 3:

Please provide a detailed explanation as to why 40 variables were selected as the initial parameters.

Comment 4:

While four strategies are being considered, it seems that only Strategy 4 directly relates to clinical practice. Focusing solely on this strategy, while considering other evaluation metrics for model assessment, would make the paper simpler and more useful to the reader.

Comment 5:

It appears that the variables included in the final model are primarily those known to be important in clinical practice. The performance of a simple logistic regression model with these variables as explanatory variables should be considered. A model that includes interaction terms between variables might also be a potential candidate. The usefulness of this model lies in its ability to resolve the interpretability issue associated with machine learning. If this model does not perform significantly worse, the proposed machine learning model may be considered inferior in terms of interpretability.

Comment 6: On Minimizing the False Negative Rate

The model is designed to minimize the false negative rate to 1-3%, but it is necessary to ensure that this model provides information beyond clinical diagnosis. There needs to be a discussion on how this is evaluated and what considerations were made regarding this point.

Comment 7:

Displaying SHAP scores to represent the interpretability of the model would be useful.

Comment 8:

The text in the figures is too small, making it difficult to read the figures. Improvements are needed to enhance figure readability.

**Reviewer**	**2**
**Name**	**Malek, Sorayya**
**Affiliation**	**University of Malaya Faculty of Science, ISB, Bioinformatics**
**Date**	**28-Nov-2024**
**COI**	**No competing interests**

---

1. Introduction

Lack of Justification for Algorithm Selection:

The rationale for choosing CatBoost, XGBoost, and LightGBM over simpler or alternative models like neural networks or logistic regression is not explained.

There is no mention of why these algorithms align with the dataset characteristics or the clinical goals.

Limited Context of Explainability:

The importance of explainability in predictive models for clinical use is not discussed, reducing the perceived relevance of the model for clinicians.

2. Literature Review

Incomplete Comparison with Prior Models:

The paper does not critically compare the strengths and limitations of existing IHCA prediction models or explain how SCARS-2 advances them.

There is no discussion of whether SCARS-2 achieves better calibration, explainability, or ease of use compared to previous models.

No Discussion on Handling Missing Data in Previous Studies:

Missing data handling strategies from prior studies are not reviewed, leaving the current approach uncontextualized.

Explainability in Prior Models Not Addressed:

The paper does not explore whether explainable AI techniques were incorporated into other models, missing an opportunity to highlight potential improvements with SCARS-2.

3. Methods

Absence of Data Summary Statistics:

Basic descriptive statistics (e.g., distributions, proportions of survival outcomes, extent of missing data) are missing. This makes it hard to evaluate the dataset's characteristics and potential biases.

No Flowchart for Methodology:

The workflow for preprocessing, feature selection, model training, and validation is not visually depicted, making it difficult to follow.

Data Imbalance Not Addressed:

There is no mention of whether the dataset is balanced or how class imbalance (e.g., survivor vs. non-survivor ratios) was managed. Methods like oversampling, undersampling, or SMOTE are not discussed.

Limited Transparency in Feature Selection:

The process of identifying the top 5, 10, and 20 predictors is not clearly described. It is unclear if selection was based on statistical analysis, domain knowledge, or model importance scores.

No Exploration of Missing Data Handling:

The proportion and distribution of missing data are not analyzed. It is unclear if the model's reliance on CatBoost's native handling of missing data is sufficient or if other imputation strategies were considered.

K-Fold Cross-Validation Details Missing:

The implementation of five-fold cross-validation is not detailed. For example, it is unclear whether splits were stratified, how metrics were averaged, or how overfitting risks were minimized.

Normalization and Feature Scaling:

The authors do not mention whether normalization or scaling was required for algorithms like XGBoost and LightGBM.

Unclear Pipeline for Data Preprocessing:

Steps for handling outliers, feature engineering, and other preprocessing methods are not described.

4. Results

No Analysis of Missing Data Impact:

The results do not address how missing data influenced model performance or if imputation strategies could have improved results.

Inadequate Reporting of Performance Metrics:

Essential metrics like sensitivity, specificity, precision, recall, and F1-score are missing or inadequately reported. These are critical for understanding the clinical implications of the model.

Limited Calibration Analysis:

Calibration metrics (e.g., calibration plots, Brier scores) are mentioned briefly but lack detailed analysis or visualization.

No Confidence Intervals for Metrics:

Performance metrics like AUROC are reported without confidence intervals or statistical significance, making it difficult to assess their robustness.

Threshold-Based Analysis Missing:

The paper does not evaluate performance at clinically relevant decision thresholds. Metrics like false positive rate (FPR) or false negative rate (FNR) at specific thresholds are missing.

Limited Explanation of Feature Subsets:

While the authors test the top 5, 10, and 20 predictors, they do not explain how these subsets were selected or validated. The impact of these subsets on model generalizability is not explored.

5. Discussion

Lack of Explainability Analysis:

The discussion does not address the importance of explainability for clinical adoption. Techniques like SHAP or LIME could have been used to make the model more interpretable.

No Consideration of Data Bias:

The paper does not explore whether missing data, imbalanced classes, or specific data characteristics may have biased the results.

Generalizability Issues:

The discussion lacks an in-depth exploration of how well the model might generalize to populations outside Sweden or in different healthcare systems.

No Trade-off Analysis:

The trade-offs between sensitivity and specificity are not discussed, which are critical for understanding the clinical risks of false positives and false negatives.

Overlooked Simpler Models:

There is no comparison with simpler interpretable models like logistic regression to justify the added complexity of machine learning approaches

6. Conclusion

Overstatement of Contributions:

The conclusion emphasizes SCARS-2 as a significant advancement but does not acknowledge its reliance on standard methodologies or its limitations in clinical adoption.

Limited Discussion of Practical Use:

There is no detailed discussion of how the model could be integrated into clinical workflows or its potential for real-world implementation.

Overall Summary of Key Missing Components

Performance Metrics:

Sensitivity, specificity, PPV, NPV, F1-score, MCC, and calibration metrics like Brier scores should be included.

Threshold-based metrics for clinically relevant decision points are missing.

Explainability:

The use of SHAP values, LIME, or other explainable AI techniques is absent, reducing the interpretability of the model.

Generalizability:

External validation and subgroup analyses are necessary to ensure the model's applicability across diverse populations.

Data Preprocessing:

Handling of missing data, class imbalance, normalization, and feature selection is not adequately detailed.

Transparency:

The methodology lacks clarity, including a flowchart and descriptive statistics.

VERSION 1 - AUTHOR RESPONSE

Response to Reviewer 1 (Dr. Sho Komukai, Osaka University):

Thank you for your thoughtful feedback. We have reorganized the manuscript to improve clarity and flow. Specifically, we have emphasized the clinical applicability of our model by restructuring the sections to present key findings in a more logical sequence. This restructuring enhances overall readability and ensures the model's performance and interpretability are clearly highlighted. We have also included new figures to illustrate the model's predictive power and interpretability, improving the manuscript's clinical relevance. Below are our responses to each of your comments:

Comment 1: It appears that cases where ROSC occurred within 15 minutes are included in the analysis population. However, considering the purpose of this prediction model, I feel it

would be more useful to exclude such cases and build a prediction model for the population where ROSC did not occur within 15 minutes.

Response: Thank you for this insightful comment. We included cases where ROSC occurred within the first 15 minutes to capture the critical early resuscitation period, which significantly impacts survival outcomes. Early interventions, such as defibrillation, are known to improve survival rates in this window (Spearpoint et al., 2000, reference 13 in the updated manuscript). Excluding these cases would limit the model's ability to predict real-world outcomes, as patients who achieve early ROSC still require intensive care for long-term survival. It would also reduce its utility in guiding resource allocation and clinical decisions during initial resuscitation efforts. By including these cases, SCARS-2 remains clinically relevant and provides a more comprehensive tool for decision-making during the early resuscitation phase.

Comment 2: Regarding Missing Data: Even in clinical settings, future data collection for diagnosis should assume that measurements can be made without missing data. A more detailed discussion is needed on whether missing data holds any significance in the prediction context. It should also be clarified in the paper how missing values were handled within the model. If it is assumed that the missing data is "Missing at Random," methods such as data imputation or inverse probability weighting for missing data should be applied. In particular, the missing rate for the initial rhythm is 26.3%, which is extremely concerning.

Response: Thank you for this thoughtful feedback. While it is ideal to assume complete data collection, missing data is inevitable in real-world clinical settings, especially given the high-pressure nature of emergency care. In this study, rather than relying on imputation methods that might introduce bias, we leveraged machine learning algorithms like XGBoost and CatBoost, which are designed to handle missing data natively. This approach ensures our model remains robust and generalizable in clinical settings. Although 26.3% of the initial rhythm data was missing, this did not significantly affect model performance. However, we acknowledge the potential implications of missing data, and we plan to explore advanced imputation methods such as multiple imputations or inverse probability weighting in future work to evaluate their impact on model accuracy and clinical generalizability.

Comment 3: Please provide a detailed explanation as to why 40 variables were selected as the initial parameters.

Response: Thank you for your question. We started with 393 candidate predictors and performed feature importance analysis to narrow down the list. The 30-40 variables selected were those that meaningfully contributed to model performance while balancing predictive accuracy and simplicity. This selection process minimized overfitting and ensured the model remained clinically relevant and efficient. **(Incorporated- Line number: 101-105)**

Comment 4: While four strategies are being considered, it seems that only Strategy 4 directly relates to clinical practice. Focusing solely on this strategy, while considering other evaluation metrics for model assessment, would make the paper simpler and more useful to the reader.

Response: Thank you for your comment. We agree that Strategy 4, which focuses on minimizing false negatives, is particularly relevant to clinical practice. While we initially presented multiple strategies (AUROC, calibration, and F1-score) to demonstrate the model's robustness, we recognize the importance of emphasizing FNR reduction. In response to suggestion, we have rearranged the manuscript to better highlight the clinical implications of Strategy 4. The discussion has been streamlined to focus more on this strategy, with a clearer explanation of its relevance to real-world clinical decision-making, while still maintaining reference to the other strategies for a comprehensive evaluation of model performance.

Comment 5: It appears that the variables included in the final model are primarily those known to be important in clinical practice. The performance of a simple logistic regression model with these variables as explanatory variables should be considered. A model that includes interaction terms between variables might also be a potential candidate. The usefulness of this model lies in its ability to resolve the interpretability issue associated with machine learning. If this model does not perform significantly worse, the proposed machine learning model may be considered inferior in terms of interpretability.

Response: Thank you for your comment. We compared the performance of logistic regression with CatBoost using both all features and the top features identified by CatBoost. When using all features, CatBoost outperformed logistic regression (AUROC: 0.914 vs. 0.885), and when restricted to the top 15 features, CatBoost still maintained the highest AUROC of 0.910, compared to 0.887 for logistic regression. These results show that CatBoost captures complex interactions between variables, which are essential for accurate survival prediction. While logistic regression is interpretable, CatBoost also provides transparency through SHAP plots, which offer clear insights into the model's decision-making, making it more interpretable for clinical use. **(Incorporated- Line number: 260-272, Figure 3, and 4, Supplementary Figure 5)**

Comment 6: On Minimizing the False Negative Rate: The model is designed to minimize the false negative rate to 1-3%, but it is necessary to ensure that this model provides information beyond clinical diagnosis. There needs to be a discussion on how this is evaluated and what considerations were made regarding this point.

Response: Thank you for your insightful comment. While we did not optimize explicitly for a 1-3% false negative rate (FNR), we previously conducted a detailed analysis within this range, considering its importance for clinical relevance. However, in response to your suggestion, we have now expanded our analysis across a broader range of thresholds. This allows the model to better balance sensitivity and specificity, ensuring it is adaptable to different clinical scenarios and risk tolerances. We believe this broader analysis provides more actionable insights for clinicians**. (Incorporated – Line numbers: 243-246, Figure 2(c))**

Comment 7: Displaying SHAP scores to represent the interpretability of the model would be useful.

Response: Thank you for your helpful suggestion. We've included SHAP plots in the revised manuscript, which clearly illustrate feature importance and show how each predictor contributes

to the model's predictions. We believe this enhances the model's interpretability and will assist clinicians in making more informed decisions. **(Incorporated- Figure 3, and 4)**

Comment 8: The text in the figures is too small, making it difficult to read the figures. Improvements are needed to enhance figure readability.

Response: Thank you for pointing this out. We have updated the figures in the revised manuscript by increasing the text size and improving clarity to enhance readability. We hope these adjustments make the figures more accessible and easier to interpret.

Response to Reviewer 2 (Dr. Sorayya Malek, University of Malaya Faculty of Science):

Thank you very much for your constructive and thoughtful comments. We greatly appreciate the time and effort you took to review our manuscript. Your feedback has been invaluable in improving the clarity, depth, and rigor of the paper. In response to your concerns, we have made several revisions, including additional analyses, figures, and a more detailed discussion. We hope that the changes made adequately address your suggestions, and we would like to address each of your points below:

Comment 1: Introduction

Lack of Justification for Algorithm Selection: The rationale for choosing CatBoost, XGBoost, and LightGBM over simpler or alternative models like neural networks or logistic regression is not explained.

There is no mention of why these algorithms align with the dataset characteristics or the clinical goals.

Response: Thank you for your valuable comment. We selected CatBoost, XGBoost, and LightGBM for several important reasons. These algorithms are particularly well-suited for clinical datasets due to their ability to handle categorical variables, missing data, and complex interactions between features. In comparison, simpler models like logistic regression assume linear relationships and often require extensive feature engineering to achieve meaningful performance in non-linear, high-dimensional datasets. Additionally, neural networks, while powerful, typically require larger datasets to perform effectively and can suffer from lower interpretability, which is a critical concern in clinical settings where understanding the decision-making process is essential. On the other hand, models like CatBoost, XGBoost, and LightGBM offer a balanced combination of predictive power and interpretability. This makes them more appropriate for clinical applications, where both accuracy and transparency are crucial for informed decision-making. **(Incorporated- Line number: 42-50)**

Limited Context of Explainability: The importance of explainability in predictive models for clinical use is not discussed, reducing the perceived relevance of the model for clinicians.

Response: Thank you for raising this concern. We understand that explainability is a critical factor when implementing predictive models in clinical practice. In response to your comment, we have

taken steps to emphasize the importance of model transparency. In the revised manuscript, we have included SHAP plots to visualize and explain the decision-making process of the models, which provides clear insights into how each feature contributes to the model's predictions. This feature enhances the interpretability of the model and ensures that clinicians can trust and understand the reasoning behind each prediction. We believe that this added clarity makes the model more suitable for clinical use, where transparency and trust in automated decision support systems are essential for optimal patient care**. (Incorporated- Line number: 275-289, 337-346, Figure 3, and 4)**

Comment 2: Literature Review

Incomplete Comparison with Prior Models: The paper does not critically compare the strengths and limitations of existing IHCA prediction models or explain how SCARS-2 advances them.

There is no discussion of whether SCARS-2 achieves better calibration, explainability, or ease of use compared to previous models.

Response: Thank you for your comment. We have expanded the discussion on how SCARS-2 compares with prior IHCA prediction models, focusing on its improvements in calibration, explainability, and robustness in handling missing data. While earlier models primarily relied on traditional methods such as manual feature engineering and imputation, SCARS-2 utilizes CatBoost, a more advanced algorithm that can handle missing data natively, which ensures more robust and accurate predictions, especially in clinical settings where missing data is common. Additionally, SCARS-2 shows improvements in calibration, making it a more reliable tool for real-world use. The paper now more clearly emphasizes how SCARS-2 builds on and improves upon these prior models, addressing key limitations such as data incompleteness and the lack of transparency. **(Incorporated- Line number: 18-23, 348-368)**

No Discussion on Handling Missing Data in Previous Studies: Missing data handling strategies from prior studies are not reviewed, leaving the current approach uncontextualized.

Response: Thank you for this important suggestion. In the revised manuscript, we have included a more thorough review of missing data handling strategies. Most previous IHCA prediction models relied on imputation methods to handle missing data, which can introduce bias and reduce reliability. In contrast, SCARS-2 uses CatBoost, which can natively handle missing values without the need for imputation. This capability significantly enhances the robustness of SCARS-2, especially in clinical settings where missing data is often inevitable. We believe this distinction is an important improvement over prior models and have emphasized it in the revised manuscript. **(Incorporated- Line number: 321-329)**

Explainability in Prior Models Not Addressed: The paper does not explore whether explainable AI techniques were incorporated into other models, missing an opportunity to highlight potential improvements with SCARS-2.

Response: Thank you for this valuable point. We have added a discussion regarding the lack of explainability in prior IHCA prediction models, which typically did not incorporate explainable AI techniques. SCARS-2 improves upon these models by integrating SHAP plots, a widely used explainability tool, to provide clear insights into how the model makes its predictions. This transparency is crucial for clinical use, where understanding and trusting the model's decision-making process is essential. By including SHAP plots, SCARS-2 enhances clinician confidence and facilitates the use of AI in high-stakes decision-making environments**. (Incorporated- Line number: 348-368)**

Comment 3: Methods

Absence of Data Summary Statistics: Basic descriptive statistics (e.g., distributions, proportions of survival outcomes, extent of missing data) are missing. This makes it hard to evaluate the dataset's characteristics and potential biases.

Response: Thank you for pointing this out. We have included basic descriptive statistics in Table 1, which includes the distributions of key variables, the proportions of survival outcomes, and the extent of missing data. This will provide a clearer understanding of the dataset's characteristics and any potential biases.

No Flowchart for Methodology: The workflow for preprocessing, feature selection, model training, and validation is not visually depicted, making it difficult to follow.

Response: Thank you for your valuable suggestion. To enhance clarity, we have reorganized the methods section and included a flowchart in the supplementary materials **(Supplementary Figure 1)** that visually outlines the entire methodology, from preprocessing and feature selection to model training and validation. This revision aims to provide a more transparent and easier-to-follow depiction of our workflow.

Data Imbalance Not Addressed: There is no mention of whether the dataset is balanced or how class imbalance (e.g., survivor vs. non-survivor ratios) was managed. Methods like oversampling, undersampling, or SMOTE are not discussed.

Response: Thank you for raising this important issue. We addressed data imbalance by incorporating class weights during model training. This approach ensures that the model considers the underrepresented classes appropriately, helping mitigate the impact of any imbalance in the dataset. **(Incorporated- Line number: 146-153)**

Limited Transparency in Feature Selection: The process of identifying the top 5, 10, and 20 predictors is not clearly described. It is unclear if selection was based on statistical analysis, domain knowledge, or model importance scores.

Response: Thank you for your insightful comment. The top 5, 10, and 20 predictors were selected based on model importance scores, derived from CatBoost's built-in feature importance mechanism. These subsets were further validated using cross-validation to ensure their robustness. The final analysis focuses on the top 15 predictors, as performance improvements

beyond this subset were minimal, based on validation results. To show an even distribution, results were presented for the 5, 10, 15, and 20 predictors. A plot showing how the AUC varies with different subsets of predictors has been included in the revised manuscript **(Figure 1(b))**, and detailed results for all subsets are provided in the supplementary materials **(Supplementary Figure 4).**

No Exploration of Missing Data Handling: The proportion and distribution of missing data are not analyzed. It is unclear if the model's reliance on CatBoost's native handling of missing data is sufficient or if other imputation strategies were considered.

Response: Thank you for this important point. In our study, we relied on CatBoost's native handling of missing data, as it can naturally manage missing values during model training. This is particularly beneficial in clinical datasets, where missing data is common. While other imputation strategies could be considered, we found that CatBoost's approach provided sufficient handling for our analysis. We plan to explore additional imputation methods in future work to assess their impact on model performance.

K-Fold Cross-Validation Details Missing: The implementation of five-fold cross-validation is not detailed. For example, it is unclear whether splits were stratified, how metrics were averaged, or how overfitting risks were minimized.

Response: Thank you for your feedback. We have now provided additional details regarding the five-fold cross-validation in the manuscript. We used stratified five-fold cross-validation to maintain the distribution of classes across all folds. We also describe how metrics were averaged across folds for more reliable performance evaluation. To minimize overfitting, we employed early stopping, halting training when no improvement was observed in validation performance. These measures ensure the robustness of our model. **(Incorporated- Line number: 126-133)**

Normalization and Feature Scaling: The authors do not mention whether normalization or scaling was required for algorithms like XGBoost and LightGBM.

Response: We appreciate your comment. Normalization was applied where necessary, as recommended for optimal performance. However, it is worth noting that the models used (XGBoost, LightGBM, and CatBoost) are robust to variations in feature scale. Therefore, additional scaling was not required in most cases. **(Incorporated- Line Number:121-124, Supplementary Figure 1)**

Unclear Pipeline for Data Preprocessing: Steps for handling outliers, feature engineering, and other preprocessing methods are not described.

Response: Thank you for pointing this out. We have now provided more details regarding the data preprocessing pipeline. While explicit outlier handling was not performed, we note that the models used (XGBoost, LightGBM, and CatBoost) are robust to outliers, which reduces the need for explicit outlier removal. However, we acknowledge that handling outliers could improve model performance and plan to investigate this further in future work.

Comment 4: Results

No Analysis of Missing Data Impact: The results do not address how missing data influenced model performance or if imputation strategies could have improved results.

Response: We appreciate this feedback. In the revised manuscript, we clarify that both CatBoost and XGBoost handle missing data internally without the need for separate imputation, which is a key feature of these algorithms. However, we recognize that further exploration of missing data and its potential effects could enhance the understanding of model performance.

Inadequate Reporting of Performance Metrics: Essential metrics like sensitivity, specificity, precision, recall, and F1-score are missing or inadequately reported. These are critical for understanding the clinical implications of the model.

Response: Thank you for pointing this out. We have now included a more comprehensive set of performance metrics, including sensitivity, specificity, precision, recall, and F1-score, which are evaluated across various decision thresholds. This addition provides a clearer understanding of the model's clinical performance and its ability to balance different aspects of prediction. **(Incorporated- Line number: 233-250, 307-319)**

Limited Calibration Analysis: Calibration metrics (e.g., calibration plots, Brier scores) are mentioned briefly but lack detailed analysis or visualization.

Response: We acknowledge the need for a more thorough calibration analysis. In response, we have added calibration plots and Brier scores to the manuscript to provide a detailed evaluation of model calibration. These metrics offer a better understanding of how well the predicted probabilities align with the actual outcomes, ensuring the model's reliability in clinical use. **(Incorporated- Figure 2(b), Line number:228-231)**

No Confidence Intervals for Metrics: Performance metrics like AUROC are reported without confidence intervals or statistical significance, making it difficult to assess their robustness.

Response: We appreciate your suggestion. Confidence intervals for AUROC and calibration metrics are now provided in the manuscript. This addition allows for a more robust evaluation of the model's performance and ensures transparency in the statistical reliability of our results. **(Incorporated- Figure 2(a), Line number:225-228)**

Threshold-Based Analysis Missing: The paper does not evaluate performance at clinically relevant decision thresholds. Metrics like false positive rate (FPR) or false negative rate (FNR) at specific thresholds are missing.

Response: We agree that threshold-based analysis is important for clinical decision-making. As such, we have added a detailed threshold-based analysis in the revised manuscript. This includes an evaluation of sensitivity, specificity, and false negative rate (FNR) at various clinically

relevant decision thresholds, providing a deeper understanding of the trade-offs between performance metrics at these thresholds. **(Incorporated- Figure 2(c), Line number:233-251)**

Explanation of Feature Subsets: While the authors test the top 5, 10, and 20 predictors, they do not explain how these subsets were selected or validated. The impact of these subsets on model generalizability is not explored.

Response: We appreciate this comment and have now clarified the process of feature selection. The top 5, 10, and 20 predictors were selected based on model importance scores, as determined by the CatBoost algorithm. These subsets were validated using cross-validation to ensure their robustness. We focus on the top 15 predictors in the final analysis, as performance improvement beyond this subset was minimal, based on validation data. To ensure full transparency, detailed results for all subsets are included in the supplementary materials (**Supplementary Figure 4**). We also acknowledge that the choice of predictor subset has an impact on model generalizability, and we propose exploring this further in future work.

Comment 5: Discussion

Lack of Explainability Analysis: The discussion does not address the importance of explainability for clinical adoption. Techniques like SHAP or LIME could have been used to make the model more interpretable.

Response: Thank you for raising this important point. We fully recognize that explainability is crucial for clinical adoption. To address this, we have now included SHAP plots in the revised manuscript, which provide transparent insights into the model's decision-making process. **(Incorporated- Figure 3, and 4)**

No Consideration of Data Bias: The paper does not explore whether missing data, imbalanced classes, or specific data characteristics may have biased the results.

Response: We appreciate your comment on this. In the revised manuscript, we discuss the potential data biases that could have affected the model's performance, including class imbalance and missing data. We have also clarified the steps we took to mitigate these biases, such as using class weights to address the class imbalance and leveraging CatBoost's native handling of missing data, which ensures more reliable performance despite incomplete data. **(Incorporated- Line number: 110-114, 146-153)**

Generalizability Issues: The discussion lacks an in-depth exploration of how well the model might generalize to populations outside Sweden or in different healthcare systems.

Response: We understand the importance of generalizability and have now included a more detailed discussion on this. We acknowledge that the model was trained on data from Sweden and propose that future validation studies should assess its performance across diverse populations and healthcare systems. This will help ensure the model's robustness and applicability in a broader clinical context. **(Incorporated- Line number: 377-387)**

No Trade-off Analysis: The trade-offs between sensitivity and specificity are not discussed, which are critical for understanding the clinical risks of false positives and false negatives.

Response: Thank you for pointing this out. In the revised manuscript, we have expanded the analysis of sensitivity and specificity at various thresholds to explore the trade-offs between these two metrics. This analysis provides a clearer understanding of how the model balances the risks of false positives and false negatives, which is essential for clinical decision-making. **(Incorporated- Line number: 307-318)**

Overlooked Simpler Models: There is no comparison with simpler interpretable models like logistic regression to justify the added complexity of machine learning approaches

Response: We appreciate your suggestion. A comparison with logistic regression has been included in the revised manuscript. Our results show that machine learning models like SCARS-2 offer significant advantages over simpler models, such as logistic regression, particularly in capturing complex interactions and patterns in the data. This allows SCARS-2 to provide more accurate predictions, justifying the added complexity of using advanced machine learning techniques in this context. **(Incorporated- Line number: 260-272)**

Comment 6: Conclusion

Overstatement of Contributions: The conclusion emphasizes SCARS-2 as a significant advancement but does not acknowledge its reliance on standard methodologies or its limitations in clinical adoption.

Response: We appreciate this feedback and have made revisions to ensure a more balanced representation of SCARS-2's contributions. The conclusion now highlights that SCARS-2 builds upon established methodologies, and we have explicitly acknowledged its current limitations, including its early-stage development and the need for further validation before clinical adoption. This provides a clearer perspective on the model's current status and future potential. **(Incorporated- Line number: 377-387)**

Limited Discussion of Practical Use: There is no detailed discussion of how the model could be integrated into clinical workflows or its potential for real-world implementation. Overall Summary of Key Missing Components

Response: Thank you for pointing this out. In response, we have expanded the discussion to address the practical integration of SCARS-2 into clinical workflows. Specifically, we describe how SCARS-2 could assist in decision-making within emergency settings, enhancing real-time predictions. We also acknowledge that successful real-world implementation will require further steps, including seamless integration with clinical systems and real-time data validation, which we view as important future directions. **(Incorporated- Line number: 377-387)**

Performance Metrics: Sensitivity, specificity, PPV, NPV, F1-score, MCC, and calibration metrics like Brier scores should be included. Threshold-based metrics for clinically relevant decision points are missing.

Response: We thank you for this valuable suggestion. We have now included additional performance metrics in the manuscript, such as sensitivity, specificity, positive predictive value (PPV), negative predictive value (NPV), F1-score, and Matthew's correlation coefficient (MCC), along with calibration metrics like Brier scores. Moreover, threshold-based metrics have been added to provide clinically relevant decision points, ensuring a more comprehensive evaluation of the model's performance. (Incorporated- Line number: 233-251, 307-318)

Explainability: The use of SHAP values, LIME, or other explainable AI techniques is absent, reducing the interpretability of the model.

Response: We recognize the importance of model explainability, especially in clinical settings. To address this, we have incorporated SHAP plots in the revised manuscript, which illustrate the impact of individual features on the model's predictions. This enhances the model's interpretability, making it more transparent and clinically relevant. **(Incorporated- Line number: 337-346, Figure 3 and Figure 4, Supplementary Figure 5)**

Generalizability: External validation and subgroup analyses are necessary to ensure the model's applicability across diverse populations.

Response: We appreciate your comment regarding generalizability. In response, we have expanded the discussion to emphasize the need for external validation and subgroup analyses to evaluate the model's performance across different populations and healthcare settings. This step is essential for ensuring the robustness and broad applicability of SCARS-2. **(Incorporated- Line number: 377-387)**

Data Preprocessing: Handling of missing data, class imbalance, normalization, and feature selection is not adequately detailed.

Response: We understand the importance of providing clarity on the preprocessing steps. In the revised manuscript, we have provided a more detailed description of the data preprocessing methods, including how missing data were handled (using CatBoost's native handling of missing values), class imbalance was addressed (via class weights), normalization was applied where necessary, and feature selection was based on model importance scores. This ensures greater transparency in the modelling process. **(Incorporated- Line number: 116-113, 146-153)**

Transparency: The methodology lacks clarity, including a flowchart and descriptive statistics.

Response: We have taken steps to enhance the clarity and transparency of the methodology. A flowchart illustrating the entire process from preprocessing and feature selection to model training and validation has been added to the supplementary materials. Additionally, we now

provide a table with basic descriptive statistics of the dataset, offering a clear overview of the data and methods used. (. **(Incorporated- Supplementary Figure 1)**

VERSION 2 - REVIEW

**Reviewer**	**1**
**Name**	**Komukai, Sho**
**Affiliation**	**Osaka University**
**Date**	**14-Feb-2025**
**COI**	

Thank you for giving me the opportunity to review this paper again. I believe that the content of the paper has been appropriately revised overall. However, I feel that the description regarding missing data is insufficient. While I understand that CatBoost and XGBoost handle missing data within their algorithms using decision trees, other analyses, such as logistic regression, should appropriately address missing data through imputation or other methods. Since one of the objectives of this study is to compare different analytical methods, it is necessary to ensure that the conditions for comparison are equal across methods. Therefore, the manuscript should clearly state how missing data were handled.

Additionally, in the response to the comments, it is stated that "Although 26.3% of the initial rhythm data was missing, this did not significantly affect model performance." However, which results substantiate this claim? It is unclear and should be explicitly clarified.

**Reviewer**	**2**
**Name**	**Malek, Sorayya**
**Affiliation**	**University of Malaya Faculty of Science, ISB, Bioinformatics**
**Date**	**03-Feb-2025**
**COI**	

This study provides a promising application of machine learning for IHCA survival prediction, but major revisions are required before it can be considered for publication. The lack of justification, improper handling of missing data, absence of clinical score comparisons, missing calibration analysis, and lack of external validation significantly limit its clinical impact. Until these issues are addressed, the real-world clinical applicability of this ML model remains uncertain.

Major Concerns and Recommendations

1. Lack of Justification for Using Machine Learning Models

The authors claim that logistic regression has limitations, but they do not provide sufficient justification for why ML models are necessary for this task. The study lacks a comparative analysis demonstrating why traditional models fail in capturing survival risk.

Without a clear justification, the ML approach may not be needed if simpler models achieve similar performance.

• Provide a rigorous justification for ML by comparing model performance on interaction effects, non-linearity, and high-dimensional data representation.

• Perform a direct comparison between logistic regression and ML models on key predictive factors.

2. Poor Handling of Missing Data

The study relies on CatBoost's native handling of missing data but does not assess how missingness affects model performance. Several critical features have high missingness, including:

• Pulse on arrival (65.2% missing)

• Initial rhythm (23.6% missing)

• Ejection fraction (31.4% missing)

There is no analysis of whether missing data is completely at random (MCAR), at random (MAR), or not at random (MNAR), and no sensitivity analysis was performed to evaluate alternative imputation techniques.

• Conduct a missing data pattern analysis to determine whether missingness is MCAR, MAR, or MNAR.

• Perform a sensitivity analysis using different missing data handling techniques, including multiple imputation and mean/mode imputation.

• Report whether different missing data handling strategies affect model performance.

3. Lack of Comparison with Established Clinical Risk Scores

The authors only compare their model to logistic regression, but they fail to compare it against widely used clinical risk scores, such as:

• GO-FAR (Good Outcome Following Attempted Resuscitation Score)

• NEWS (National Early Warning Score)

• SAPS II / APACHE (ICU mortality scores)

These established scores are already used in hospital settings, and without this comparison, it is unclear if ML provides a meaningful improvement.

• Benchmark the ML model against GO-FAR, NEWS, SAPS II, or APACHE to establish clinical relevance.

• Compare AUROC, sensitivity, specificity, and PPV/NPV between ML models and clinical scoring systems.

4. No Proper Calibration Comparison Between ML and Traditional Methods

The authors report a calibration plot and a Brier Score (0.11) for CatBoost, but they do not provide calibration comparisons for logistic regression or other ML models (XGBoost, LightGBM).

Additionally, there is no Hosmer-Lemeshow test to evaluate logistic regression calibration. Without these analyses, it remains unclear whether ML models provide better-calibrated predictions compared to traditional approaches.

• Report Brier Scores and calibration plots for all models.

• Conduct a Hosmer-Lemeshow test for logistic regression.

5. No Decision Curve Analysis (DCA) to Assess Clinical Benefit

The study lacks Decision Curve Analysis (DCA), which is essential for assessing whether ML-based predictions provide clinical benefit over existing models. A high AUROC does not necessarily translate into clinical usefulness, and DCA helps quantify net clinical benefit.

• Perform DCA to evaluate whether ML models improve decision-making in real-world clinical settings.

6. No Statistical Testing to Prove ML Superiority Over Logistic Regression

The authors report AUROC values, stating that CatBoost (0.91) outperforms logistic regression (0.885). However, they do not conduct statistical significance testing (e.g., DeLong’s test) to confirm that this difference is statistically significant rather than due to random variation.

• Conduct DeLong’s test or equivalent statistical methods to verify if the difference in AUROC between logistic regression and ML models is significant.

7. Lack of Robust Validation and Feature Selection

The authors use an 80-20 train-test split to evaluate their machine learning models but do not mention using k-fold cross-validation, which is standard for robust evaluation. Without k-fold cross-validation, the results are prone to split bias, and the reported performance metrics may not generalize to new datasets. Additionally, the authors do not specify whether the train-test split was stratified, which is critical given the class imbalance.

Regarding feature selection, the study used model-based rankings to select the top 40 predictors and ultimately reduced the set to 15 features. However, the cutoff for selecting 40 features appears arbitrary, and no alternative feature selection techniques (e.g., LASSO or

Recursive Feature Elimination) were explored. There is also no discussion on the clinical relevance of excluded or retained features.

VERSION 2 - AUTHOR RESPONSE

Reviewer#1
Dr. Sho Komukai, Osaka University

Comments to the Author:
Thank you for giving me the opportunity to review this paper again. I believe that the content of the paper has been appropriately revised overall.

However, I feel that the description regarding missing data is insufficient. While I understand that CatBoost and XGBoost handle missing data within their algorithms using decision trees, other analyses, such as logistic regression, should appropriately address missing data through imputation or other methods. Since one of the objectives of this study is to compare different analytical methods, it is necessary to ensure that the conditions for comparison are equal across methods. Therefore, the manuscript should clearly state how missing data were handled.
Additionally, in the response to the comments, it is stated that "Although 26.3% of the initial rhythm data was missing, this did not significantly affect model performance." However, which results substantiate this claim? It is unclear and should be explicitly clarified.

> **Response**: We appreciate the reviewer's comment on handling missing data. As mentioned, CatBoost and XGBoost handle missing data internally, while logistic regression used imputation. A comparison of model performance with and without imputation is provided in Table 2. For CatBoost, AUC remained stable (0.9135 vs. 0.9136), while XGBoost and LightGBM showed slight drops in AUC with imputation, but sensitivity improved slightly (e.g., XGBoost: from 0.7046 to 0.7121). The Brier score exhibited minimal changes for CatBoost and LightGBM, with XGBoost showing a slight increase with imputation. Sensitivity increased slightly for LightGBM (from 0.7137 to 0.7165), while specificity slightly decreased (from 0.9287 to 0.9250), reflecting the typical trade-off with imputation. These results indicate that imputation had minimal impact on performance, with some models showing improvements in sensitivity**. [Table 2 on page number 15. Lines: 181-196 (page number 14)]**

Reviewer# 2
Dr. Sorayya Malek, University of Malaya Faculty of Science

Comments to the Author:
This study provides a promising application of machine learning for IHCA survival prediction, but major revisions are required before it can be considered for publication. The lack of justification, improper handling of missing data, absence of clinical score comparisons, missing calibration analysis, and lack of external validation significantly limit its clinical impact. Until these issues are addressed, the real-world clinical applicability of this ML model remains uncertain.

Major Concerns and Recommendations:

1. Lack of Justification for Using Machine Learning Models
The authors claim that logistic regression has limitations, but they do not provide sufficient justification for why ML models are necessary for this task. The study lacks a comparative analysis demonstrating why traditional models fail in capturing survival risk.
Without a clear justification, the ML approach may not be needed if simpler models achieve similar performance.

- Provide a rigorous justification for ML by comparing model performance on interaction effects, non-linearity, and high-dimensional data representation.
- Perform a direct comparison between logistic regression and ML models on key predictive factors.

Response: We appreciate the reviewer's comment regarding the justification for using machine learning (ML) models over logistic regression. Over the course of seven years, we have rigorously tested our dataset using logistic regression, support vector machines (SVM), tree-based ensemble methods, deep learning frameworks, and boosting techniques. Through these extensive experiments, which involved hundreds of thousands of models, we consistently found that boosted models significantly outperform logistic regression in predictive accuracy and robustness.

To provide clear empirical justification, we have now conducted an updated comparative analysis that evaluates multiple models across key performance metrics. We have added Table 2, which presents a direct comparison of CatBoost, XGBoost, LightGBM, and logistic regression under different conditions (with and without multiple imputation).

Key Findings from Table 2: **[Table 2 on page number 15. Lines: 181-208 (page number 14-15)]**

Boosted models (CatBoost, XGBoost, LightGBM) consistently achieve higher AUC values (~0.913) compared to logistic regression (AUC = 0.8976).

Logistic regression underperforms in key areas such as sensitivity (0.6985 vs. 0.7117 in CatBoost), specificity (0.9157 vs. 0.9287 in CatBoost), and F1-score (0.7425 vs. 0.7626 in CatBoost).

The Brier score (a measure of calibration) is lower for boosting models (~0.1028), indicating better probability estimation compared to logistic regression (0.1122).

The performance gap remains even after applying multiple imputation, confirming that logistic regression is consistently inferior.

This analysis demonstrates that logistic regression struggles with capturing complex interactions, non-linear relationships, and high-dimensional feature spaces, where boosted models excel. Given these findings, machine learning models are essential for accurately predicting survival risk in this dataset.

We have now included these results in Table 2 and revised the manuscript to clarify why ML models are necessary.

2. Poor Handling of Missing Data

The study relies on CatBoost's native handling of missing data but does not assess how missingness affects model performance. Several critical features have high missingness, including:

• Pulse on arrival (65.2% missing)
• Initial rhythm (23.6% missing)
• Ejection fraction (31.4% missing)

There is no analysis of whether missing data is completely at random (MCAR), at random (MAR), or not at random (MNAR), and no sensitivity analysis was performed to evaluate alternative imputation techniques.

• Conduct a missing data pattern analysis to determine whether missingness is MCAR, MAR, or MNAR.
• Perform a sensitivity analysis using different missing data handling techniques, including multiple imputation and mean/mode imputation.
• Report whether different missing data handling strategies affect model performance.

Response: We sincerely appreciate the reviewer's insightful comments regarding the handling of missing data. Sudden cardiac arrest presents unique challenges for predictive modelling due to the chaotic nature of the event. The entire sequence—from circulatory collapse to the arrival of the emergency team—often unfolds in a matter of minutes, and witnesses are frequently in shock, leading to uncertainty regarding both significant and non-significant details. Consequently, missing data in key variables (such as pulse on arrival, initial rhythm, and ejection fraction) is common and reflects the reality of sudden cardiac arrest scenarios.

Added related matter: **[Table 2 on page number 15. Lines: 181-208 (page number 14-15)]**

1. Missing Data Pattern Analysis

To address the missing data issue, we conducted Little's MCAR test, which indicated that the missing data is not completely at random (MCAR), but more likely to be missing at random (MAR) or missing not at random (MNAR). Specifically, we found that pulse on arrival (65.2% missing) is likely MNAR, as it depends on whether emergency responders were able to measure it. On the other hand, ejection fraction (31.4% missing) is frequently missing due to the unavailability of echocardiography at the time of the event.

2. Sensitivity Analysis Using Different Imputation Techniques

We performed a sensitivity analysis using various imputation techniques, including mean imputation, multiple imputation by chained equations (MICE), and no imputation. The results presented in Table 2 indicate that boosted models (CatBoost, XGBoost, and LightGBM) consistently outperformed logistic regression across all key metrics.

3. Justification for Not Imputing Missing Data

Based on our previous experience (Sultanian et al., 2023: https://pubmed.ncbi.nlm.nih.gov/38774371/), we found that imputing missing data during model training often leads to overestimated model performance, particularly when the test set is also imputed. However, models trained with imputed data did not perform well when deployed in real-world environments, where missing data is intrinsic and cannot be imputed. This led us to revise our approach and exclude imputation during model training to better reflect the conditions encountered in clinical practice. This adjustment is supported by findings from Hessulf et al. (2023: https://pubmed.ncbi.nlm.nih.gov/36773348/), which also emphasized the limitations of imputation in scenarios with high missingness.

While models trained with imputed data showed improved performance in training and validation, they performed poorly when deployed in real-world settings where missing data is inevitable. This discrepancy highlights the challenge of imputing missing data during model training, as it introduces an unrealistic advantage that is not present in clinical scenarios.

We believe this approach provides a more realistic representation of how our models would perform in real-world clinical settings, and we have revised the manuscript accordingly to clarify this rationale.

3. Lack of Comparison with Established Clinical Risk Scores
The authors only compare their model to logistic regression, but they fail to compare it against widely used clinical risk scores, such as:
• GO-FAR (Good Outcome Following Attempted Resuscitation Score)
• NEWS (National Early Warning Score)
• SAPS II / APACHE (ICU mortality scores)
These established scores are already used in hospital settings, and without this comparison, it is unclear if ML provides a meaningful improvement.
• Benchmark the ML model against GO-FAR, NEWS, SAPS II, or APACHE to establish clinical relevance.
• Compare AUROC, sensitivity, specificity, and PPV/NPV between ML models and clinical scoring systems.

Response: We appreciate the reviewer's suggestion to compare our model with other risk scores. However, we specifically developed our model for a scenario where there are no risk scores. As evident in manuscript, our model is intended to be used in the general setting, on any ward in the hospital, after cardiac arrest has occurred, and more specifically after 15 minutes of resuscitation. The other mentioned models, on the other hand have the following validation settings:

- GO-FAR is used to predict the likelihood of survival with good neurologic outcome after in-hospital cardiac arrest (IHCA), but at the time of hospital admission for adult patients.
- NEWS (National Early Warning Score) is used to assess and track the clinical condition of adult patients (aged 16 years or more) to detect clinical deterioration and identify patients at risk of adverse events. Therefore, NEWS is not to be compared with our model.

- SAPS II / APACHE (ICU mortality scores): These scoring systems are used specifically for critically ill patients admitted to Intensive Care Units (ICUs), which constitutes a minority of our patient population, in addition to predicting mortality at admission to the ICU.

Hence, while we recognize that comparing models is a good idea and these are relevant models, the scenario, patient population and purpose of these models differ too much from our model, which makes the comparison undesirable. We will however, due to this comment, make our model publicly available via GitHub (https://github.com/Vibha190685/Predicting-30-day-survival-after-in-hospital-cardiac-arrest/tree/main) so that anyone can use it for their comparison. [**Lines: 341-369 (page number 21-22)**]

4. No Proper Calibration Comparison Between ML and Traditional Methods
The authors report a calibration plot and a Brier Score (0.11) for CatBoost, but they do not provide calibration comparisons for logistic regression or other ML models (XGBoost, LightGBM).
Additionally, there is no Hosmer-Lemeshow test to evaluate logistic regression calibration. Without these analyses, it remains unclear whether ML models provide better-calibrated predictions compared to traditional approaches.
• Report Brier Scores and calibration plots for all models.
• Conduct a Hosmer-Lemeshow test for logistic regression.

Response: We appreciate the reviewer's suggestion to provide a calibration comparison between machine learning (ML) models and traditional logistic regression. We have addressed this as follows:

1. **Brier Scores**: We calculated Brier Scores for all models (CatBoost: 0.1028, XGBoost: 0.1035, LightGBM: 0.1027, Logistic Regression: 0.1122). The results show that ML models generally perform better than logistic regression. [**Incorporated: Supplementary Figure 2**]

2. **Calibration Plots**: We have included calibration plots for all models. These plots further support the superior calibration of the ML models compared to logistic regression. [**Incorporated: Supplementary Figure 2**]

3. **Hosmer-Lemeshow Test**: We performed the Hosmer-Lemeshow test for logistic regression. Most folds (1, 2, 3, 5) showed a good fit (p-value ≥ 0.05), but Fold 4 showed poor calibration ($p < 0.05$). **[Lines: 198-208 (page number 15), supplementary section C]**

5. No Decision Curve Analysis (DCA) to Assess Clinical Benefit
The study lacks Decision Curve Analysis (DCA), which is essential for assessing whether ML-based predictions provide clinical benefit over existing models. A high AUROC does not necessarily translate into clinical usefulness, and DCA helps quantify net clinical benefit.
• Perform DCA to evaluate whether ML models improve decision-making in real-world clinical settings.

Response: We appreciate the reviewer's suggestion to include Decision Curve Analysis (DCA) to assess the clinical benefit of the models. In response, we have performed DCA and plotted the results for all models. The DCA curve, which is provided as **Supplementary Figure 3**, shows that all ML models (CatBoost, XGBoost, and LightGBM)

consistently outperform logistic regression in terms of net clinical benefit. This highlights the superior decision-making potential of ML models over traditional approaches in real-world clinical settings. [**Incorporated: Supplementary Figure 3**]

6. No Statistical Testing to Prove ML Superiority Over Logistic Regression
The authors report AUROC values, stating that CatBoost (0.91) outperforms logistic regression (0.885). However, they do not conduct statistical significance testing (e.g., DeLong's test) to confirm that this difference is statistically significant rather than due to random variation.
• Conduct DeLong's test or equivalent statistical methods to verify if the difference in AUROC between logistic regression and ML models is significant.

Response: We appreciate the reviewer's thoughtful suggestion to include statistical testing for the comparison of AUROC values. In response, we have performed paired t-tests to assess the statistical significance of the differences in AUROC between Logistic Regression and the machine learning models (CatBoost, LightGBM, and XGBoost). The results are as follows:

- CatBoost vs Logistic Regression: t = 17.428, p = 0.000 (significant)
- LightGBM vs Logistic Regression: t = 11.813, p = 0.000 (significant)
- XGBoost vs Logistic Regression: t = 4.433, p = 0.011 (significant)

As all p-values are below 0.05, it confirms that the differences in AUROC are statistically significant, with the ML models demonstrating superior performance compared to Logistic Regression. We thank the reviewer for emphasizing the importance of this analysis. **[Lines: 198-208 (page number 15)]**

7. Lack of Robust Validation and Feature Selection
The authors use an 80-20 train-test split to evaluate their machine learning models but do not mention using k-fold cross-validation, which is standard for robust evaluation. Without k-fold cross-validation, the results are prone to split bias, and the reported performance metrics may not generalize to new datasets. Additionally, the authors do not specify whether the train-test split was stratified, which is critical given the class imbalance.

Response: We appreciate the reviewer's comment. As initially stated, we performed experiments using an 80/20 train-test split with stratified sampling due to class imbalance. During the optimization process, we applied 5-fold cross-validation on the 80% training data, and this procedure was repeated 5 times to ensure robust evaluation. As per the reviewer's suggestion, we have now re-evaluated all results using 5-fold cross-validation with stratified sampling and have updated the manuscript accordingly. The results from this new approach are consistent with the initial findings, confirming the stability of our model performance. [**Lines: 114-120 (page number 9), Lines: 143-147 (page number 10)**]]

Regarding feature selection, the study used model-based rankings to select the top 40 predictors and ultimately reduced the set to 15 features. However, the cutoff for selecting 40 features appears arbitrary, and no alternative feature selection techniques (e.g., LASSO or Recursive Feature Elimination) were explored. There is also no discussion on the clinical relevance of excluded or retained features.

Response: We completely agree with the reviewer that feature selection and dimensionality reduction could be done with structured methodologies, and we have done so on many instances over the years. However, RFE and LASSO generally results in the same top features being selected as using the built-in importance measures of tree based models (with some exceptions (Conditional variable importance for random forests - PubMed)). Ultimately, we have simplified our modelling, for computation reasons, to directly go for selecting the top features according to the tree-based model. Then the question arises whether retraining on a reduced number of features would result in a different model being the best model, and that is the reason why we first reduce to a relatively large subset of the original features *40 in this study, before retraining again to select the top ones, and then evaluate the performance of this reduced model. This approach has been evaluated by our group over many years, including comparisons with RFE, LASSO, etc / and it has been non/inferior in model performance, albeit saving much computational time. [**Lines: 305-319 (page number 20)**]

VERSION 3 - REVIEW

**Reviewer**	**2**
**Name**	**Malek, Sorayya**
**Affiliation**	**University of Malaya Faculty of Science, ISB, Bioinformatics**
**Date**	**13-Apr-2025**
**COI**	

The authors have addressed all my previous concerns, including justification for feature selection, handling of class imbalance, and validation strategy. The methodology is now clear and supported by appropriate metrics. I find the revised manuscript suitable for publication.
